# Assembly of Polyiodide Networks with Cu(II) Complexes of Pyridinol-Based Tetraaza Macrocycles

**DOI:** 10.1021/acs.inorgchem.1c02967

**Published:** 2021-12-22

**Authors:** Álvaro Martínez-Camarena, Matteo Savastano, Salvador Blasco, Estefanía Delgado-Pinar, Claudia Giorgi, Antonio Bianchi, Enrique García-España, Carla Bazzicalupi

**Affiliations:** † ICMol, Department of Inorganic Chemistry, University of Valencia, C/Catedrático José Beltrán 2, 46980 Paterna, Spain; ‡ Department of Chemistry “Ugo Schiff”, University of Florence, Via della Lastruccia 3-13, 50019 Sesto Fiorentino, Italy; § Department of Chemistry, CQC, University of Coimbra, P3004-535 Coimbra, Portugal

## Abstract

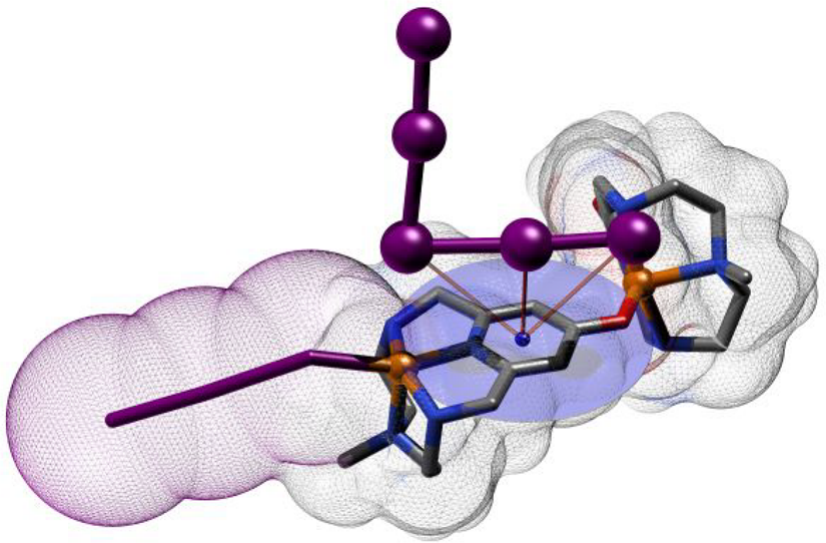

Polyiodide networks are currently of great practical interest for the preparation of new electronic materials. The participation of metals in the formation of these networks is believed to improve their mechanical performance and thermal stability. Here we report the results on the construction of polyiodide networks obtained using Cu(II) complexes of a series of pyridinol-based tetraazacyclophanes as countercations. The assembly of these crystalline polyiodides takes place from aqueous solutions on the basis of similar structural elements, the [CuL]^2+^ and [Cu(H_–1_L)]^+^ (L = **L2**, **L2-Me**, **L2-Me**_**3**_) complex cations, so that the peculiarities induced by the increase of N-methylation of ligands, the structural variable of ligands, can be highlighted. First, solution equilibria involving ligands and complexes were analyzed (potentiometry, NMR, UV–vis, ITC). Then, the appropriate conditions could be selected to prepare polyiodides based on the above complex cations. Single-crystal XRD analysis showed that the coordination of pyridinol units to two metal ions is a prime feature of these ligands, leading to polymeric coordination chains of general formula {[Cu(H_–1_L)]}_*n*_^*n*+^ (L = **L2-Me**, **L2-Me**_**3**_). In the presence of the I^–^/I_2_ couple, the polymerization tendency stops with the formation of [(CuL)(CuH_–1_L)]^3+^ (L = **L2-Me**, **L2-Me**_**3**_) dimers which are surrounded by polyiodide networks. Moreover, coordination of the pyridinol group to two metal ions transforms the surface charge of the ring from negative to markedly positive, generating a suitable environment for the assembly of polyiodide anions, while N-methylation shifts the directional control of the assembly from H-bonds to I···I interactions. In fact, an extended concatenation of iodine atoms occurs around the complex dimeric cations, the supramolecular I···I interactions become shorter and shorter, fading into stronger forces dominated by the orbital overlap, which is promising for effective electronic materials.

## Abstract 2

Polyiodides with high iodine density are generated by Cu(II) complexes of pyridinol-based tetraazacyclophanes. Direct coordination of iodine atoms to Cu(II), anion−π interactions with electron-poor aromatic surfaces, and shift of the directional control of assembly from H-bonds to I···I interactions, governed by N-methylation, are the main elements leading to enhanced iodine chaining and strengthening of I···I contacts.

## Introduction

Small polyazacycloalkanes have aroused a great interest since the earliest years of macrocyclic chemistry, as the convergent arrangement of their donor atoms allows for strong complexation of many transition-metal ions. The early inclusion of N-heterocyclic, aromatic groups into their macrocyclic structures introduced several additional properties related to ligand rigidity, modulation of binding characteristics, implementation of π-interactions, and activation of mechanisms, based on the absorption and/or the emission properties of N-heteroaromatic groups, to sense and report the interactions with guest species.^1^

**Figure 1:**
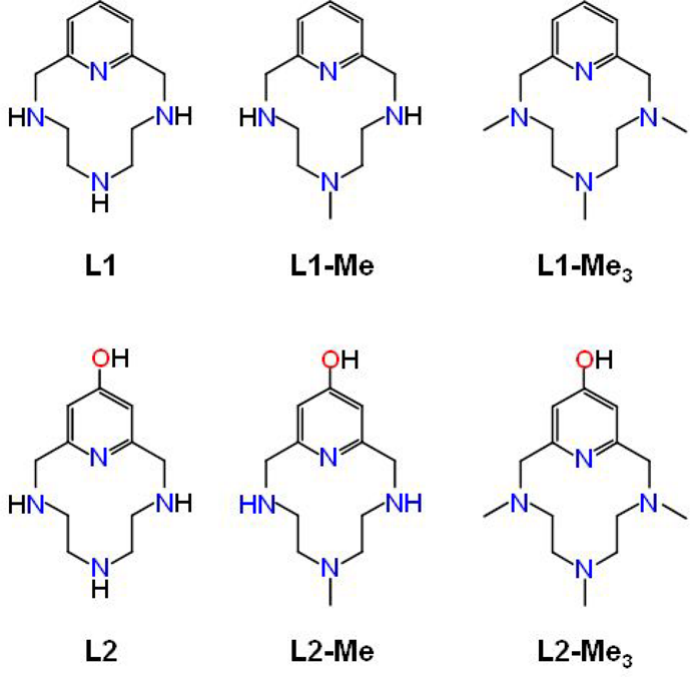
Ligands drawing.

A noteworthy member of this class of macrocyclic ligands is 3,6,9-triaza-1-(2,6)-pyridinacyclodecaphane (**L1**, Figure 1) which, in the three decades since its appearance,^2^ has given rise to a large family of derivatives that have been investigated for applications in many areas, including, among others, pharmaceutical and biomedical sectors,^3−17^ catalysis,^18−23^ and chemosensing^24−27^ as well as in the preparation of polybromides^28^ and polyiodides^29,30^ featuring iodine-dense three-dimensional networks that might open up new perspectives in crystal engineering and in the design of solid-state conductors as well as in the preparation of new chemicals with antimicrobial activity.^31−33^

A considerable part of these applications involved metal complexes and exploited free coordination positions on metal ions as active sites: **L1** and many of its derivatives do not contain enough donor atoms to fulfill metal coordination spheres. We have recently shown,^30^ for instance, that metal centers in Cu(II) complexes of **L1** and its methylated derivatives **L1-Me** and **L1-Me**_**3**_ (Figure 1) can aid in the formation of particular polyiodide networks that are assembled from simple tectons, such as I^–^ and I_2_, passing through complex I_3_^–^, I_5_^–^, and I_7_^–^ anions and the unusual I_8_^2–^ species which connects two Cu(II) centers.

Many metals can participate in the formation of iodine chains.^34^ They are thought to enhance the mechanical performance and the thermal stability of polyiodides and, as recently shown,^35^ to modulate their conduction properties. I···I interactions, in addition to being an opportunity for semantic considerations,^36^ are the connectors that can make solid-state conduction feasible and efficient based on a Grotthuss-like mechanism,^37,38^ which requires the preservation of orbital overlap along the iodine chains, a phenomenon favored by high iodine density.^37,39,40^ Nonetheless, it has been shown that the physical properties, that is, electrical conductivity and thermal stability, of polyiodide chains are also affected by the supramolecular interactions they experience in the crystals.^41^ These details form a basis of information that can aid in the design of high-performance polyiodide-based solid-state conductors, which are of currently great practical interest for the development of new devices, especially for the preparation of solar cells^42,43^ and batteries.^44,45^

The progressive N-methylation of the ligand, from **L1** to **L1-Me** and **L1-Me**_**3**_, offered the opportunity to observe the effect of the transition to less polar environments around the metal centers.^30^ The loss of H-bond donor groups causes a shift from the hydrogen-bond domain to that of I···I interactions, in terms of the main forces participating in the organization of the crystalline phases, that is accompanied by the greater participation of Cu(II) ions in the stabilization of the polyiodide network.

Modifications of the electron density of the pyridine group could be another tool for fine-tuning the metal coordination properties of these ligands and the propagation of their electronic properties to the surrounding polyiodide networks either by direct connection to the metal ions (polyiodide coordination) and by proximity to surfaces of modified electron density. Nevertheless, only few examples of pyridine-substituted derivatives of **L1**, **L1-Me**, and **L1-Me**_**3**_, bearing both electron-donating and electron-withdrawing groups, have been reported, so far.^29,46−48^ Among these, only **L2-Me**_**3**_ (Figure 1) has been used for the preparation of polyiodides, in the absence of metal ions, leading to the [H_2_**L2-Me**_**3**_(I_7_)_2_] compound featuring diprotonated ligand molecules segregated into boxes formed by couples of tripodal I_7_^–^ anions.^29^ Analysis of its crystal structure led us to believe that the formation of these rare I_7_^–^ anions is due to the capacity of (H_2_**L2-Me**_**3**_)^2+^ to act as a supramolecular templating agent, molding the heptaiodide anion around itself. This arrangement of polyiodide units, despite forming a dense iodine lattice (iodine number I_*N*_ = 0.589 for [H_2_**L2-Me**_**3**_(I_7_)_2_]),^49^ does not allow for the formation of an extended iodine network which is a mandatory requirement for solid state conduction.

In this paper, we analyze the formation of Cu(II) complexes with the pyridinol ligands **L2**, **L2-Me**, and **L2-Me**_**3**_ and the extended and dense polyiodide networks we have built around these complexes. The **L2**-based ligands reproduce the progressive N-methylation of the **L1**-based analogues, while the presence of the *para* −OH group introduces two additional factors: (i) the possibility of intermolecular interactions (formation of oligomeric complexes or coordination polymers) via coordinative/hydrogen bonding of deprotonated/protonated −OH groups and (ii) alteration of the electronic properties of the pyridine ring brought about by this *para* substituent. With respect to the latter, the *para* −OH group increases the electron density on the pyridine ring,^48^ a phenomenon that would seem to generate an adverse environment for the stabilization of polyiodide anions. Nevertheless, metal coordination to the pyridinol nitrogen atom facilitates the deprotonation of this −OH group and the polarization of the ring electron density toward the N-coordinated metal ion, through the stabilization of the ketone ligand form.^17^ Further depletion of ring electron density is expected to occur if the ketone oxygen is also involved in the coordination of a metal ion, thus creating favorable conditions for the stabilization of polyiodides via anion-π interactions, as actually found with the study herewith described.

## Experimental Section

### Materials

***Caution!****Solid perchlorate salts and their nonaqueous solutions are potentially explosive and should be handled in small quantities.*

All reagents were purchased from commercial sources and used as received. Solvents were of analytical grade and used without further purification. Water used for potentiometric and spectroscopic measurements was twice distilled and passed through a Millipore apparatus.

Ligands 3,6,9-triaza-1(2,6)-pyridinecyclodecaphan-1^4^-ol (**L2**),^46^ 6-(*N*-methyl)-3,6,9-triaza-1(2,6)-pyridinecyclodecaphan-1^4^-ol (**L2-Me**),^29^ and 3,6,9-tris(*N*-methyl)-3,6,9-triaza-1(2,6)-pyridinecyclodecaphan-1^4^-ol (**L2-Me**_**3**_)^29^ were synthesized as previously described.

Crystals of [Cu(H_–1_**L2-Me**)](ClO_4_)·0.716H_2_O (**1**) and [Cu(H_–1_**L2-Me**_**3**_)](ClO_4_)·H_2_O (**2**) were obtained by slow evaporation at room temperature of aqueous solutions containing equimolar quantities of ligand (**L2-Me** or **L2-Me**_**3**_) and Cu(ClO_4_)_2_·6H_2_O at pH = 7. Complex **1**: yield: 63%. Elemental analysis: calcd (%) for C_24_H_41_Cl_2_Cu_2_N_8_O_12_: C, 34.66; H, 4.97; N, 13.47. Found: C, 34.71; H, 5.09; N, 13.49. Complex **2**: yield: 74%. Elemental analysis: calcd (%) for C_28_H_48_Cl_2_Cu_2_N_8_O_11_: C, 38.62; H, 5.56; N, 12.87. Found: C, 38.57; H, 5.50; N, 12.93.

Crystals of {[(Cu**L2-Me**)(CuH_–1_**L2-Me**)I]·[(Cu**L2-Me**)(CuH_–1_**L2-Me**)]I_3_}(I_2_)(I_5_)_3_(I_7_) (**3**) and [(Cu**L2-Me**_**3**_)(CuH_–1_**L2-Me**_**3**_)I](I_2_)_2_(I_5_)_2_ (**4**) were obtained by slow diffusion, at room temperature, of aqueous solutions of complexes **1** and **2** toward iodine rich I_2_/I^–^ aqueous mixtures (I_2_:I^–^ ratio 2:1) inside H-shaped tubes. The solutions’ pH was about 6. The H-shaped tubes were loaded with 0.01 mmol of the complex on one side and an excess of the I_2_/I^–^ mixtures (I_2_ 0.1 mmol, NaI 0.05 mmol) on the other side. Crystals started being formed after about 1 week. After 4 weeks, when the solution into the tube had become uniform in color, crystals were collected by filtration, washed with water, and air dried. Complex **3**: yield: 73%. Elemental analysis: calcd (%) for C_48_H_78_Cu_4_I_28_N_16_O_4_: C, 12.14; H, 1.66; N, 4.72. Found: C, 12.21; H, 1.69; N, 4.78. Complex **4**: yield: 66%. Elemental analysis: calcd (%) for C_28_H_47_Cu_2_I_15_N_8_O_2_: C, 13.15; H, 1.85; N, 4.38. Found: C, 13.28; H, 1.88; N, 4.43.

### EMF Measurements

The potentiometric titrations were carried out at 298.1 ± 0.1 K, using NaClO_4_ 0.15 M as supporting electrolyte, in the pH range 2.5–11.0. The experimental procedure (buret, potentiometer, cell, stirrer, microcomputer, etc.) has been fully described elsewhere.^50^ The acquisition of the electromotive force (emf) data was performed with the computer program PASAT.^51,52^ The reference electrode was an Ag/AgCl electrode in saturated KCl solution. The glass electrode was calibrated as a hydrogen ion concentration probe by titration of previously standardized amounts of HCl with CO_2_-free NaOH solutions and the equivalent point determined by the Gran’s method,^53,54^ which gives the standard potential, *E*°, and the ionic product of water (p*K*_w_ = 13.73(1)). The computer program HYPERQUAD^55^ was used to fit protonation and stability constants. In all experiments, ligand concentration was about 1.0 × 10^–3^ M. In complexation experiments, the Cu(II) concentration ranged from 5.0 × 10^–4^ M to 1.0 × 10^–3^ M. Due to the very high stability of the complexes with **L2-Me** and **L2-Me**_**3**_, the polyamine 1,4,8,11-tetraazaundecane was used as a competing ligand in 1.0 × 10^–3^ M concentration. At least two titration experiments (about 100 point each) were performed for each system. The different titration curves for each system were treated both as separated curves and as merged data sets without significant variations of the determined stability constants. The final values were those obtained from the simultaneous treatment of the merged curves. The HYSS^56^ program was used to generate the distribution diagrams.

### NMR Measurements

The ^1^H and ^13^C NMR spectra were recorded on a Bruker Advance DPX300 spectrometer operating at 300.13 MHz for ^1^H and at 75.47 MHz for ^13^C. For the ^1^H spectra, the solvent signal was used as a reference standard. Adjustments to the desired pH were made using drops of DCl or NaOD solutions. The pD was calculated from the measured pH values using the correlation, pH = pD – 0.4.^57^

### UV–vis Measurements

UV–vis absorption spectra were recorded with an Agilent 8453 spectrometer. Solvents were of spectroscopic or equivalent grade. The pH of samples was measured with a Metrohm 713 pH meter, and adjustments of the hydrogen ion concentration were made with diluted HCl and NaOH solutions.

### Isothermal Titration Calorimetry

Ligand protonation enthalpies were determined in 0.15 M NaClO_4_ solution by means of a TAM III (TA Instrument) microcalorimeter equipped with a precision Lund syringe pump coupled with a 0.500 cm^3^ gastight Hamilton syringe according to a procedure already described.^30^ In a typical experiment, a NaOH solution (0.15 M, addition volumes 15 μL) was added to acidic solutions of the ligands (5 × 10^–3^ M, 1.2 cm^3^) in 0.15 M NaClO_4_. At least two titrations were performed for each system. Corrections for the heats of dilution were applied. The computer program HypCal (updated version of HypΔH)^58^ was used to calculate reaction enthalpies from calorimetric data. Ligand protonation constants used in calculations were separately determined by means of potentiometric titrations.

### Crystal Structure Determination

Blue crystals of [Cu(H_–1_**L2-Me**)](ClO_4_)·0.716H_2_O (**1**) and [Cu(H_–1_**L2-Me**_**3**_)](ClO_4_)·H_2_O (**2**) and black crystals with metallic luster of {[(Cu**L2-Me**)(CuH_–1_**L2-Me**)I]·[(Cu**L2-Me**)(CuH_–1_**L2-Me**)]I_3_}(I_2_)(I_5_)_3_(I_7_) (**3**) and [(Cu**L2-Me**_**3**_)(CuH_–1_**L2-Me**_**3**_)I](I_2_)_2_(I_5_)_2_ (**4**) were used for X-ray diffraction (XRD) analysis. A summary of the crystallographic data is reported in Table S1. The integrated intensities were corrected for Lorentz and polarization effects, and an empirical absorption correction was applied for crystals of **3** and **4**.^59^ Crystal structures of **1** and **2** were solved by using SHELXD,^60^ while SHELXT^61^ was used to solve the structures of **3** and **4**. Refinements were performed by means of full-matrix least-squares using SHELXL version 2014/7.^62^ Nonhydrogen atoms were anisotropically refined. Hydrogen atoms were introduced as riding atoms with thermal parameter calculated in agreement with the linked atom. In compound **3**, the coordinated iodide ion has been found spread over two positions, which were introduced in the calculation as I28 and I29 iodine atoms and refined with occupation factors equal to 0.65 and 0.35, respectively.

### Hirshfeld Surface Analysis

Hirshfeld surface analysis^63−65^ was performed with CrystalExplorer17.^66^

### Calculation of the ESP Surfaces

The modeling of the trimethylated ligand and its mono- and dimeric Cu(II) complexes was performed using the density functional theory computational method as well as the Becke three-parameter Lee–Yang–Parr hybrid functional.^67−69^ All the optimizations were carried out by using the Ahlrichs’ basis set def2-TZV(P)^70^ for all atoms, including copper. The effect of the polarizable solvent (water) was considered by using the default SCRF method of the polarizable continuum model.^71^ Finally, electrostatic potential (ESP) was calculated for each system by means of the Pop = (Esp,ReadRadii) command, considering the atomic radii for Cu(II) of 1.409 Å. Computations were carried out using the program Gaussian 09 C.01.^72^ and Molecular graphics and analyses were performed with gMolden^73^ and UCSF Chimera, developed by the Resource for Biocomputing, Visualization, and Informatics at the University of California, San Francisco, with support from NIH P41-GM103311.^74^

## Results and Discussion

### Ligand Protonation Equilibria

**Figure 2:**
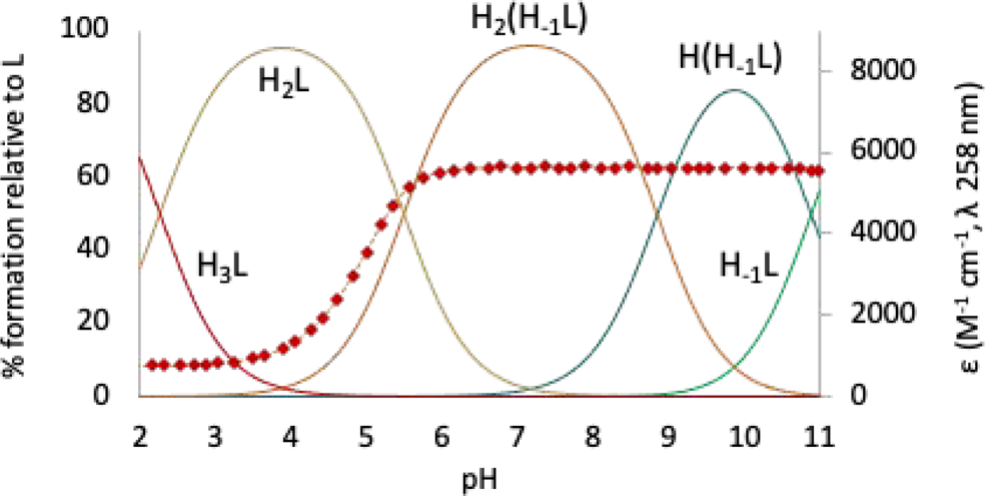
Distribution diagram of the protonated species formed by **L2** as a function of pH in aqueous solution. The extinction coefficient associated with the pyridine band in the UV–vis spectra is represented as red diamonds.

The determination of ligand protonation constants is preliminary to the determination of complexation constants and to the speciation of coordination complex systems, which are fundamental information for the preparation of polyiodide complexes. The protonation constants of the pyridinol ligands **L2**, **L2-Me**, and **L2-Me**_**3**_ were determined by means of potentiometric titrations (0.15 M NaClO_4_, 298.1 K) and are listed in Table 1 along with those previously obtained^14,16,30^ for the pyridine analogues **L1**, **L1-Me**, and **L1-Me**_**3**_ under the same experimental conditions. Distribution diagrams of the protonated species of **L2** formed as a function of pH are shown in Figure 2, while those corresponding to **L2-Me** and **L2-Me**_**3**_ can be found in Figure S1.

**Table 1:** Logarithms of Stepwise Protonation Constants of **L2**, **L2-Me**, **L2-Me_3_**, **L1**,^16^**L1-Me**,^30^ and **L1-Me_3_**,^14^ Obtained by Potentiometric Measurements in 0.15 M NaClO_4_ at 298.1 ± 0.1 K

reaction^a^	**L2**	**L2-Me**	**L2-Me**_**3**_
H_–1_L^–^ + H^+^ ⇄ H(H_–1_L)	10.89(2)^b^	11.51(2)	10.93(2)
H(H_–1_L) + H^+^ ⇄ H_2_(H_–1_L)^+^	8.85(1)	8.853(8)	8.25(2)
H_2_(H_–1_L)^+^ + H^+^ ⇄ H_2_L^2+^	5.50(1)	5.718(8)	4.91(2)
H_2_L^2+^ + H^+^ ⇄ H_3_L^3+^	2.27(3)	2.23(4)	–
log β = ∑log *K*	27.51	28.31	24.10

	**L1**	**L1-Me**	**L1-Me**_**3**_
L + H^+^ ⇄ HL^+^	10.54(1)	10.35(1)	10.88(1)
HL^+^ + H^+^ ⇄ H_2_L^2+^	7.96(1)	8.09(2)	7.37(1)
H_2_L^2+^ + H^+^ ⇄ H_3_L^3+^	1.90(1)	2.47(6)	–
log β = ∑log *K*	20.40	20.91	18.25

a H_–1_L corresponds to the ligands with deprotonated pyridinol moiety.

b Values in parentheses are standard deviations in the last significant figure.

Protonation constants of **L2** are in good agreement with literature values determined under slightly different experimental conditions (0.15 M NaCl, 298 K).^17^

As can be seen in Table 1, within the investigated pH range (2.5–11), we could determine four protonation constants for **L2** and **L2-Me** and three for **L2-Me**_**3**_, that is, protonation of these molecules is characterized by an additional equilibrium, with respect to **L1**, **L1-Me**, and **L1-Me**_**3**_, corresponding to the phenol functionalities. The fact that one less constant was found for **L2-Me**_3_, a phenomenon already observed for **L1-Me**_**3**_ can again be attributed to the significant loss of solvation experienced by the fully methylated ligands, which results in a weaker stabilization of the ammonium groups.^75^

**Figure 3:**
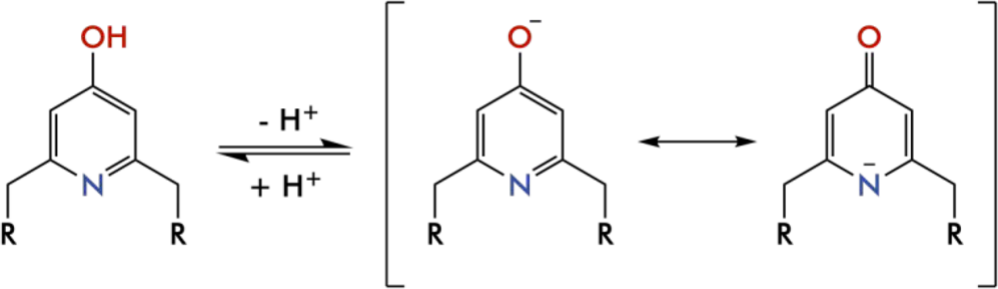
Representation of the keto–enol equilibrium determined for the pyridinol moiety.

**Figure 4:**
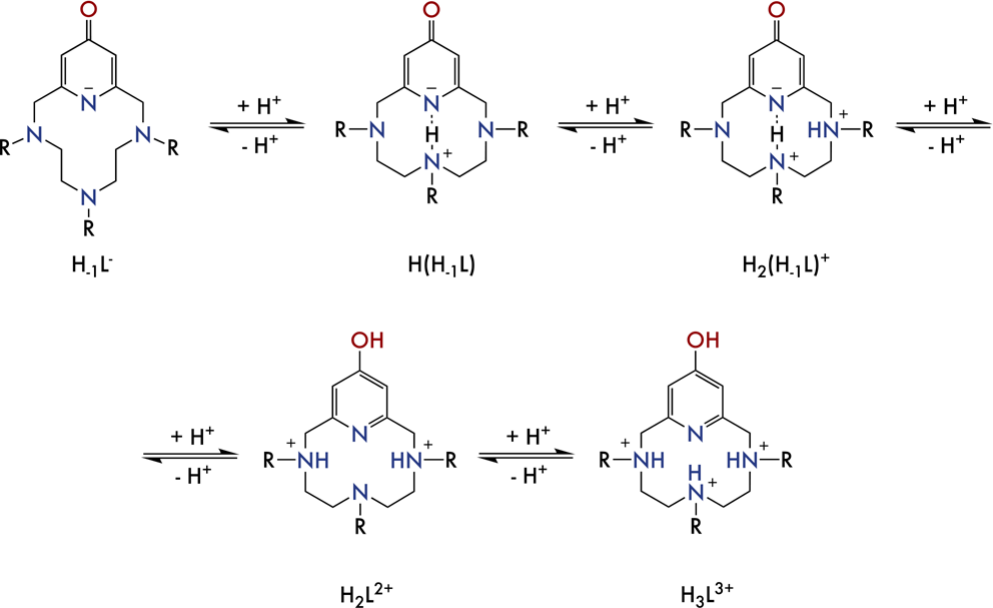
Representation of the protonation sequence proposed for the pyridinol ligands.

Another outcome of the phenol functionalities is the greater basicity of pyridinol ligands compared to pyridine ones. It is known that deprotonation of the pyridinol OH group establishes a keto–enol equilibrium that enhances the electron density on the pyridine nitrogen atom (Figure 3).^17,47,76^ This leads to a strengthening of the intramolecular hydrogen bond established between the pyridine nitrogen atom and the protonated amines of the macrocycle (Figure 4), thus increasing the value of protonation constants.

The keto–enol equilibrium and the reinforcement of the intramolecular interactions might also explain the particular acidity of this hydroxyl group. The absorbance band of the ketone oxygen (at ca. 270 nm) shows a significant decrease in intensity from pH 6 to 3, as this oxygen atom is protonated (Figures 2 and S1). Similarly, the ^1^H NMR signals of the aromatic protons shift markedly downfield from pH 6.4 to 3.6 (Figure S2). Hence, both UV and NMR measurements suggest that protonation of the ketone oxygen occurs at about pH 5, which is a particularly low pH for this group (for instance, p*K*_a_ is 11.09 for 4-hydroxypyridine, 8.72 for 3-hydroxypyridine, and 11.62 for 2-hydroxypyridine).^77^

**Figure 5:**
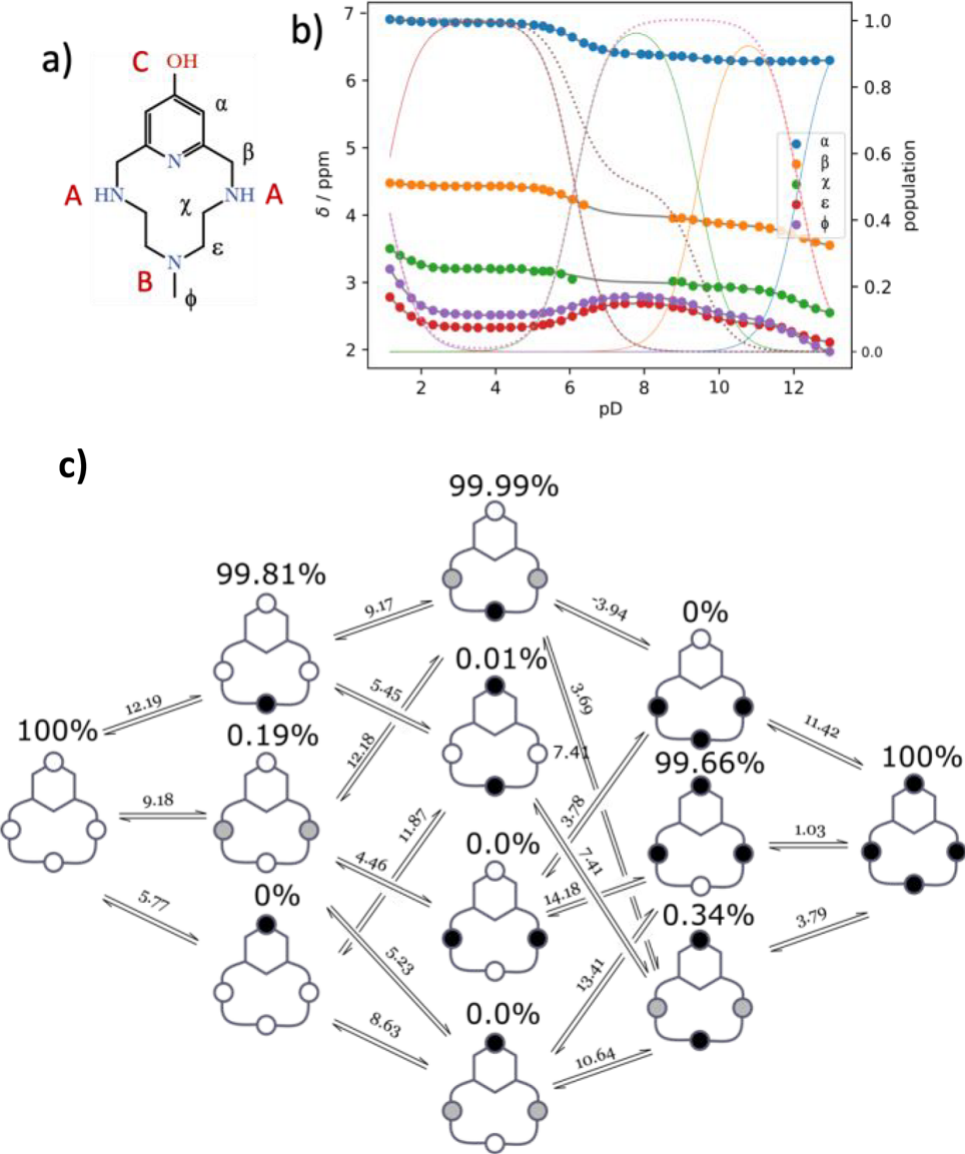
(a) Proton and protonation site labeling. (b) Experimental chemical shifts (scatter plot) and fitted chemical shifts (solid lines). (c) Summary of microstates and microconstants. Black circles represent a protonated site, gray circles represent a delocalized protonation in chemically equivalent sites, and white circles represent a nonprotonated site.

In order to fathom the protonation sequence of the macrocycles and to confirm the presence of both the keto–enol equilibrium and the intramolecular hydrogen bonds, we determined the protonation microconstants of **L2-Me** by means of pD titrations followed by ^1^H NMR. The spectra recorded for **L2-Me** at different pD values can be found in Figure S3. The raw NMR data were treated with the program GEMS based on the implementation of the Cluster Expansion Method.^78^ Representation of the variation with pD of the chemical shift of each proton signal, superimposed to the species distribution diagram of the molecules (Figure 5), allowed us to outline some interesting observations.

Starting from high pD values and moving toward the acidic zone, it can be noted that the first protonation step takes place at pD ca. 12. This reaction mainly involves the protonation center B, as shown by the upfield movement of hydrogens β, χ, ε, and, mostly, ϕ. This can be easily observed when representing the variation rate of the chemical shifts with pH, shown in Figure S4.

Unlike pyridine ligands, the second protonation of pyridinol macrocycles does not lead to a rearrangement of their protonated amino groups. The introduction of a second hydrogen ion into the molecule involves one of the two protonation centers A, while B remains protonated. This is evidenced by the upfield shift of all ^1^H NMR signals, except α. As previously discussed, the significant stability of the protonated center B can be related to its intramolecular hydrogen-bond interaction with the nitrogen atom of the pyridinol moiety in its ketone form (Figure 4). Therefore, protonation of the ketone oxygen atom is expected to imply a loss of stability which, together with the nearby H^+^ at the secondary amino group, should lead to a rearrangement of the protonated amino groups within the macrocycle, a process that actually takes place with the successive protonation step.

Indeed, the third protonation reaction leads to a major rearrangement of the protonated groups: signals ε and ϕ shift downfield, while β, χ, and, significantly, α move upfield, denoting protonation of the C and A centers and deprotonation of B, that is, with the generation of the hydroxyl form of the pyridinol moiety, the protonated center B loses its hydrogen ion in favor of the second center A, thus minimizing the repulsive interactions between positive charges. This explanation is consistent with the results of the UV titrations previously discussed.

Finally, the fourth protonation reaction, occurring at about pD 1, takes place on the only site left available, that is, B, as denoted by the fact that signals χ, ε, and ϕ shift upfield, while α and β keep unchanged.

The analysis of the raw NMR data also allowed us to determine the protonation macroconstants of the **L2-Me**, which can be found in Table S2. The resulting microstates and microconstants are summarized in Figure 5. The correction p*K*_D_ = 0.32 + 1.044 p*K*_H_ was applied to the log β obtained by the NMR studies in order to obtain the p*K*_H_ values.^57^ The obtained logarithmic constants are in reasonable agreement with those determined by the potentiometric titrations, also shown in Table S2, which supports the proposed protonation sequence.

Further characterization of protonation equilibria was performed by means of isothermal titration calorimetry (ITC) measurements to determine the corresponding enthalpy changes (Δ*H*°), which are listed in Table 2 along with the related entropic contributions (*T*Δ*S*°) derived from −*RT*ln *K* = Δ*H*° – *T*Δ*S*°. For comparison purposes, the analogous data previously determined for **L1**, **L1-Me**, and **L1-Me**_**3**_ were also included in the same table. Data for the pyridinol ligands **L2**, **L2-Me**, and **L2-Me**_**3**_ are in keeping with those for their pyridine analogues as well as with the general protonation properties of polyazacycloalkanes,^79^ especially with those of polyazacyclophanes^80^ bearing a single benzene group in the macrocyclic ring, and are consistent with the fact that the third ligand protonation occurs on the ketone oxygen atom. Indeed, as shown in Table 2, the first two protonation stages are highly exothermic reactions, while the enthalpy contribution for the third stage is much less favorable, as expected for protonation of a phenate oxygen.^81^

**Table 2:** Thermodynamic Data (kJ mol^–1^) for Protonation of **L2**, **L2-Me**, **L2-Me_3_**, **L1**,^30^**L1-Me**,^30^ and **L1-Me_3_**,^30^ Determined in 0.15 M NaClO_4_ at 298.1 ± 0.1 K

	**L2**		**L2-Me**		**L2-Me**_**3**_	
reaction^a^	Δ*H*°	*T*Δ*S*°	Δ*H*°	*T*Δ*S*°	Δ*H*°	*T*Δ*S*°
H_–1_L^–^ + H^+^ ⇄ H(H_–1_L)	–40.2(7)^b^	21.9(7)	–39.9(4)	25.8(4)	–38.8(3)	23.6(3)
H(H_–1_L) + H^+^ ⇄ H_2_(H_–1_L)^+^	–41.0(8)	9.5(8)	–41.3(4)	9.2(4)	–39.0(3)	7.5(3)
H_2_(H_–1_L)^+^ + H^+^ ⇄ H_2_L^2+^	–22.1(5)	9.2(5)	–24.7(5)	7.9(5)	–10.3(4)	16.8(4)
H_2_L^2+^ + H^+^ ⇄ H_3_L^3+^	–4.0(2)	9.0(3)	–2.1(5)	10.6(5)	–	–

	**L1**		**L1-Me**		**L1-Me**_**3**_	
	Δ*H*°	*T*Δ*S*°	Δ*H*°	*T*Δ*S*°	Δ*H*°	*T*Δ*S*°
L + H^+^ ⇄ HL^+^	–42.6(2)	17.6(2)	–39.5(5)	19.6(5)	–31.3(5)	30.8(5)
HL^+^ + H^+^ ⇄ H_2_L^2+^	–44.6(3)	0.8(3)	–48.3(7)	–2.1(7)	–39.2(6)	–2.9(6)
H_2_L^2+^ + H^+^ ⇄ H_3_L^3+^	–3.1(4)	7.7(4)	0.5(9)	13.4(9)	–	–

a H_–1_L corresponds to the ligands with deprotonated pyridinol moiety.

b Values in parentheses are standard deviations in the last significant figure.

In the fourth protonation step (not existing for **L2-Me**_**3**_), the poor proton affinity of these ligands is congruent with the almost athermic character of the protonation processes.

All protonation processes are entropically favorable: Ligand protonation causes partial charge neutralization of the bound H^+^ ions, which is accompanied by a significant desolvation effect that generates the observed favorable entropy contributions. This phenomenon is commonly observed for similar ligands.^79,80^

### Complex Formation with Cu(II)

**Figure 6:**
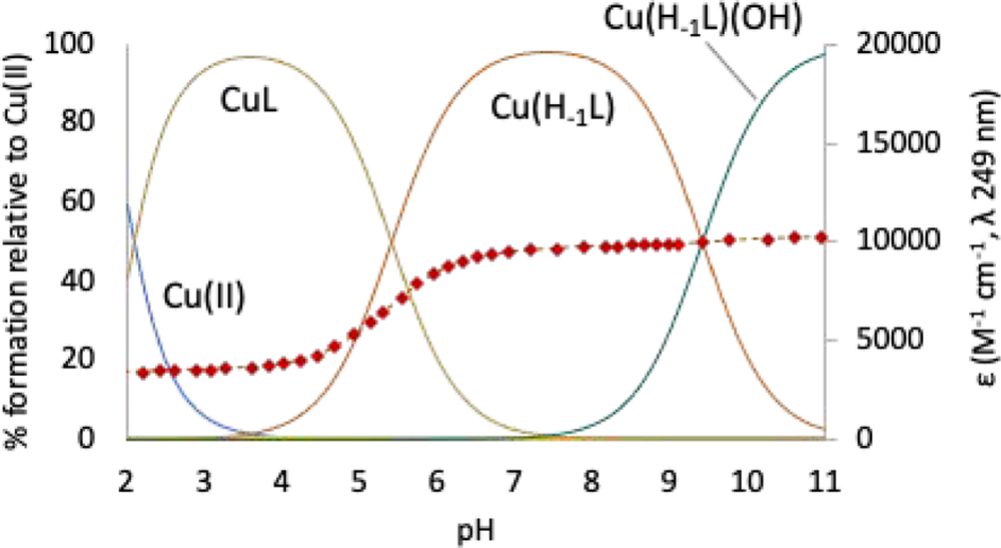
Distribution diagram of the complexes formed in the system Cu(II)/**L2**. [Cu(II)] = [**L2**] = 1 mM. Charges omitted. The extinction coefficient associated with the pyridinol band in the UV–vis spectra recorded at different pH values is represented as red diamonds.

The knowledge of the complex species formed, of their solution abundance, and of their stability under different pH conditions is basilar to establish if a complex system is appropriate for the preparation of polyiodides and, eventually, to select the best synthetic conditions. For this reason, we studied the formation of Cu(II) complexes with **L2**, **L2-Me**, and **L2-Me**_**3**_ as a function of pH by means of potentiometric titrations. The species formed and the corresponding stability constants we determined by this method are listed in Table 3, where they are compared with the corresponding data previously obtained under the same experimental conditions (0.15 M NaClO_4_, 298.1 K) for the complexes with **L1**, **L1-Me**, and **L1-Me**_**3**_. Distribution diagram of the complex species formed by **L2** is shown in Figure 6, while those corresponding to the complexes formed by **L2-Me** and **L2-Me**_**3**_ with Cu(II) can be found in Figure S5. The stability constants of **L2** complexes are in good agreement with literature values determined under slightly different experimental conditions (0.15 M NaCl, 298 K).^17^

**Table 3:** Logarithms of the Stability Constants for Cu(II) Complexes of **L2**, **L2-Me**, **L2-Me_3_**, **L1**,^30^**L1-Me**,^30^ and **L1-Me_3_**,^30^ Obtained by Potentiometric Measurements in 0.15 M NaClO_4_ at 298.1 ± 0.1 K

reaction^a^	**L2**	**L2-Me**	**L2-Me_3_**
Cu(II) + L ⇄ [CuL]^2+^	13.87(1)^b^	16.13(5)	16.4(1)
Cu(II) + H_–1_L^–^ ⇄ [Cu(H_–1_L)]^+^	19.35(3)	23.84(4)	23.32(6)
[Cu(H_–1_L)]^+^ + H_2_O ⇄ [Cu(H_–1_L)(OH)] + H^+^	–9.42(5)	–7.91(3)	–8.46(5)

	**L1**	**L1-Me**	**L1-Me_3_**
Cu(II) + L ⇄ [CuL]^2+^	17.78(2)	18.5(1)	16.44(3)
[CuL]^2+^ + H_2_O ⇄ [CuL(OH)]^+^ + H^+^	–8.68(8)	–8.73(5)	–8.53(4)
[CuL(OH)]^+^ + H_2_O ⇄ [CuL(OH)_2_] + H^+^	–10.7(1)	–10.7(2)	–11.31(8)

a H_–1_L corresponds to the ligands with deprotonated pyridinol moiety.

b Values in parentheses are standard deviations in the last significant figure.

As can be seen, these complex systems are very simple, being constituted by the three species [CuL]^2+^, [Cu(H_–1_L)]^+^, and [Cu(H_–1_L)(OH)] (L = **L2**, **L2-Me**, **L2-Me**_**3**_): H_–1_L corresponds to the ligands with deprotonated pyridinol moiety. An inspection of data in Table 3 indicates some trends of the stability constants. First of all, we can note that, with the exception of trimethylated ligands, pyridinol ligands form [CuL]^2+^ complexes less stable than pyridine ones. In particular, the difference in stability decreases from nonmethylated (Δlog *K* = 4.0 for **L1**, **L2**) to monomethylated ligands (Δlog *K* = 2.4 for **L1-M2**, **L2-Me**) and vanishes for trimethylated ones. Furthermore, the stability constants of these complexes decrease in the order **L2-Me**_**3**_ ≈ **L2-Me** > **L2** for pyridinol ligands, while the order for pyridine ones is **L1-Me** > **L1** > **L1-Me**_**3**_. Most probably, these trends are the result of opposite tendencies induced by the OH substituent on the pyridine rings and by N-methylation of ligands: (i) the electron-donating effect of OH that enhances the donor ability of the pyridinol N atom, (ii) the loss of donor ability by methylated nitrogen atoms due to the loss of M–N–H···O hydrogen bonds with water solvent molecules, and (iii) the lessening of hydration energies of methylated ligands, resulting in a lower cost for desolvation upon complexation.

However, the most notable effect caused by the OH group on Cu(II) complexation is observed when this group is deprotonated. Indeed, deprotonation of the pyridinol moiety dramatically changes the complexation behavior of these ligands by significantly increasing the stability of [Cu(H_–1_L)]^+^ complexes, relative to [CuL]^2+^ species with both pyridinolic and pyridyl nondeprotonated ligands. Such stability enhancement, which can be related to the above-discussed higher donor character of the N atom of deprotonated pyridinol ligands, is so large (up to ≈7 log units) that in the case of methylated ligands, it was necessary to adopt competition experiments (see Experimental Section) for the determination of complex stability constants. As a consequence, excluding the most acidic pH range (from 2 to 4/5), H_–1_L is the predominant form of the ligands in their Cu(II) complexes, including the very stable [Cu(H_–1_L)(OH)] species formed in the alkaline region.

In conclusion, the [Cu(H_–1_L)]^+^ complexes with **L2**, **L2-Me**, and **L2-Me**_**3**_ fulfill two fundamental requirements for being promising scaffolds for the preparation of polyiodide: (i) very high stability and (ii) presence of free (solvent occupied) coordination sites on the metal ion. While the first one ensures resistance toward demetalation processes and reduction of Cu(II) to Cu(I) by iodine, the second one favors the binding of exogenous ligands.

A final observation: The deprotonated pyridinolic oxygen atoms of [Cu(H_–1_L)]^+^ and [Cu(H_–1_L)(OH)] complexes can act as additional donor atoms favoring intermolecular contacts, an occurrence that was not observed in solution, but was found in the solid state, as shown below.

### Crystal Structures

**Figure 7:**
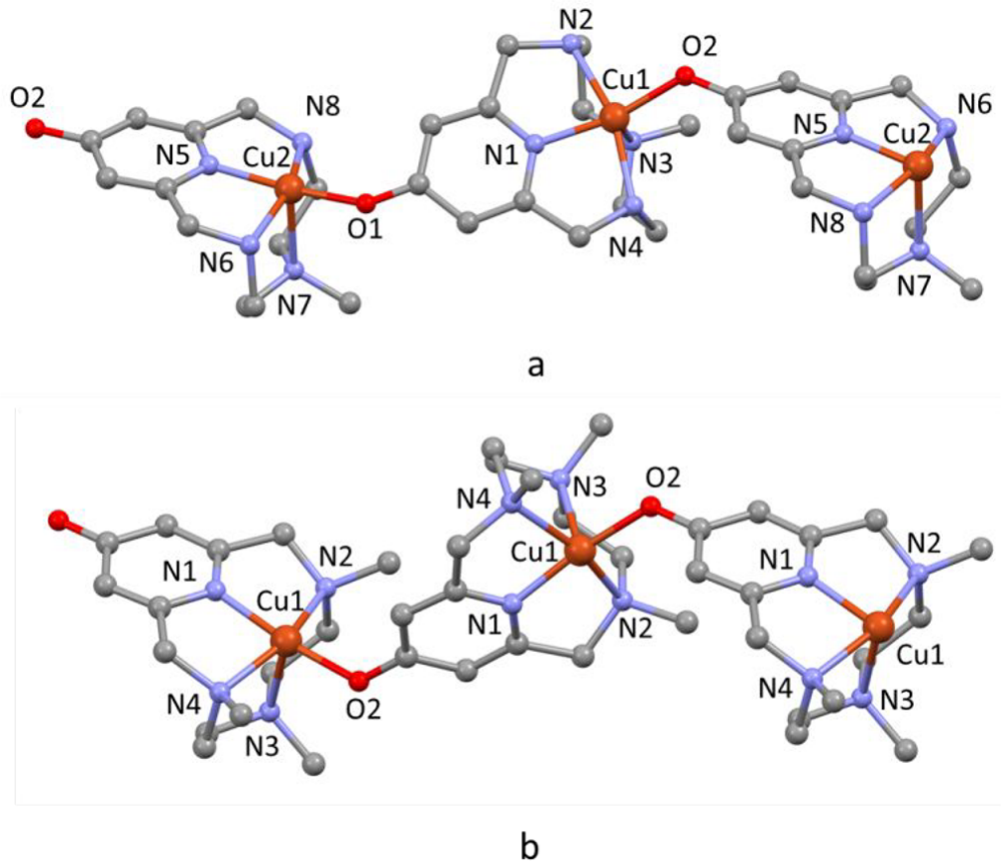
Crystal structures of (a) [Cu(H_–1_**L2-Me**)](ClO_4_)·0.716H_2_O (**1**) and (b) [Cu(H_–1_**L2-Me**_**3**_)](ClO_4_)·H_2_O (**2**).

Single crystals of [Cu(H_–1_**L2-Me**)](ClO_4_)·0.716H_2_O (**1**) and [Cu(H_–1_**L2-Me**_**3**_)](ClO_4_)·H_2_O (**2**) were subjected to XRD analysis. The asymmetric unit of **1** contains two [CuH_–1_**L2-Me**]^+^ complex cations, two ClO_4_^–^ anions (one of which is disordered), and 1.43 H_2_O molecules, while one [Cu(H_–1_**L2-Me**_**3**_)]^+^, one ClO_4_^–^ anion, and one H_2_O molecule make up the asymmetric unit of **2**. Both compounds contain polymeric chains of [Cu(H_–1_L)]^+^ (L = **L2-Me**, **L2-Me**_**3**_), extending along the [101] direction, in which the complex units are connected via coordinative interaction of the deprotonated pyridinol oxygen atoms to the Cu(II) ions of neighboring units (Figure 7a,b).

The ligands present a *cis*-folded N_4_ configuration, as already observed in similar complexes,^20,30,47,48^ so that the nitrogen atom across from the pyridinol group occupies the axial position of the axially elongated square pyramidal coordination environment of Cu(II), while the remaining three ligand nitrogen donors are located in the basal plane along with the pyridinol oxygen atom of the neighboring complex. Coordinative bond distances and angles can be found in Tables S3 and S4. The coordinative bonds in the basal plane are in the ranges 1.92–2.10 Å and 1.90–2.14 Å for **1** and **2**, respectively, while those with the apical donors are 2.23 and 2.25 Å for **1** and **2**, respectively. The coordination polymers are helically arranged in the two structures, the [Cu(H_–1_L)]^+^ units of the coordination polymers being rotated, with respect to their neighbors, 42.2° in **1** and 80.4° in **2** (as measured by the angle between the mean planes of pyridine rings). This results in a more flat and elongated helix in **1** than in **2** (helix pitch 28 Å vs 14 Å, respectively. Figure S6).

In **1**, symmetry-related polymeric chains, which appear to run in opposite directions, associate in pairs through water bridged H-bonds involving secondary nitrogen and pyridinol oxygen atoms and the formation of π–π stacking interactions between pyridinol rings coordinated to Cu2 (Figure S7): the separation between the parallel planes of these rings is 3.456 Å, the distance between ring centroids is 3.566 Å, and the displacement of the two centroid is 0.867 Å. Each pair of polymer chains is strengthened by bridging H-bonds with ClO_4_^–^ anions, which also link paired chains with neighboring analogues via direct or H_2_O-mediated H-bonds.

Unlike **1**, the polymeric chains of **2** do not give rise to direct interaction with each other, but are separated by perchlorate anions and water molecules that are located in hydrophobic pockets of the lattice surrounded by methyl or methylene groups of ligand molecules with which they form weak H-bonds.

**Scheme 1:**
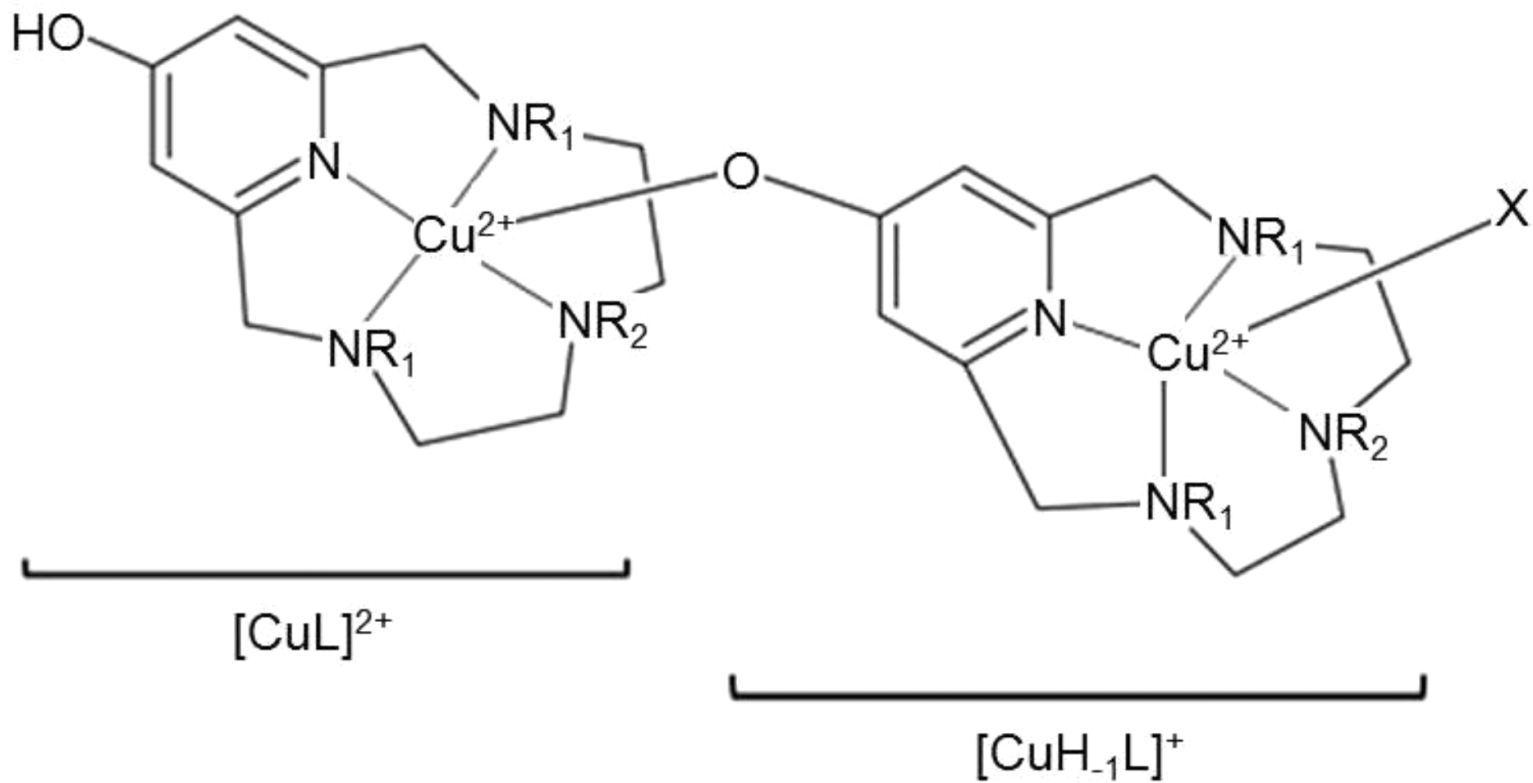
Schematic Representation of the [(CuL)(Cu(H_–1_L))X]^2+^ Dinuclear Cation (L = **L2-Me**; R_1_ = H, R_2_ = Me; L = **L2-Me_3_**; R_1_ = R_2_ = Me; X = I^–^ or I_3_^–^) Found in {[(Cu**L2-Me**)(CuH_–1_**L2-Me**)I]·[(Cu**L2-Me**)(CuH_–1_**L2-Me**)]I_3_}(I_2_)(I_5_)_3_(I_7_) (**3**) and [(Cu**L2-Me_3_**)(CuH_–1_**L2-Me_3_**)I](I_2_)_2_(I_5_)_2_ (**4**)

Cu(II) complexes with **L2**, **L2-Me**, and **L2-Me**_**3**_ were used to prepare polyiodide compounds (see Experimental Section). In the case of **L2-Me** and **L2-Me**_**3**_, we obtained crystalline samples suitable for single-crystal XRD analysis. Both contain dinuclear cations formed by the complex units (CuL)^2+^ and (CuH_–1_L)^+^ (L = **L2-Me**, **L2-Me**_**3**_), joined by coordination of (CuH_–1_L)^+^ to (CuL)^2+^ through the deprotonated pyridinol oxygen of the former. A schematic drawing of the overall [(CuL)(CuH_–1_L)X]^2+^ dinuclear cation (X = I^–^ or I_3_^–^) is shown in Scheme 1.

**Figure 8:**
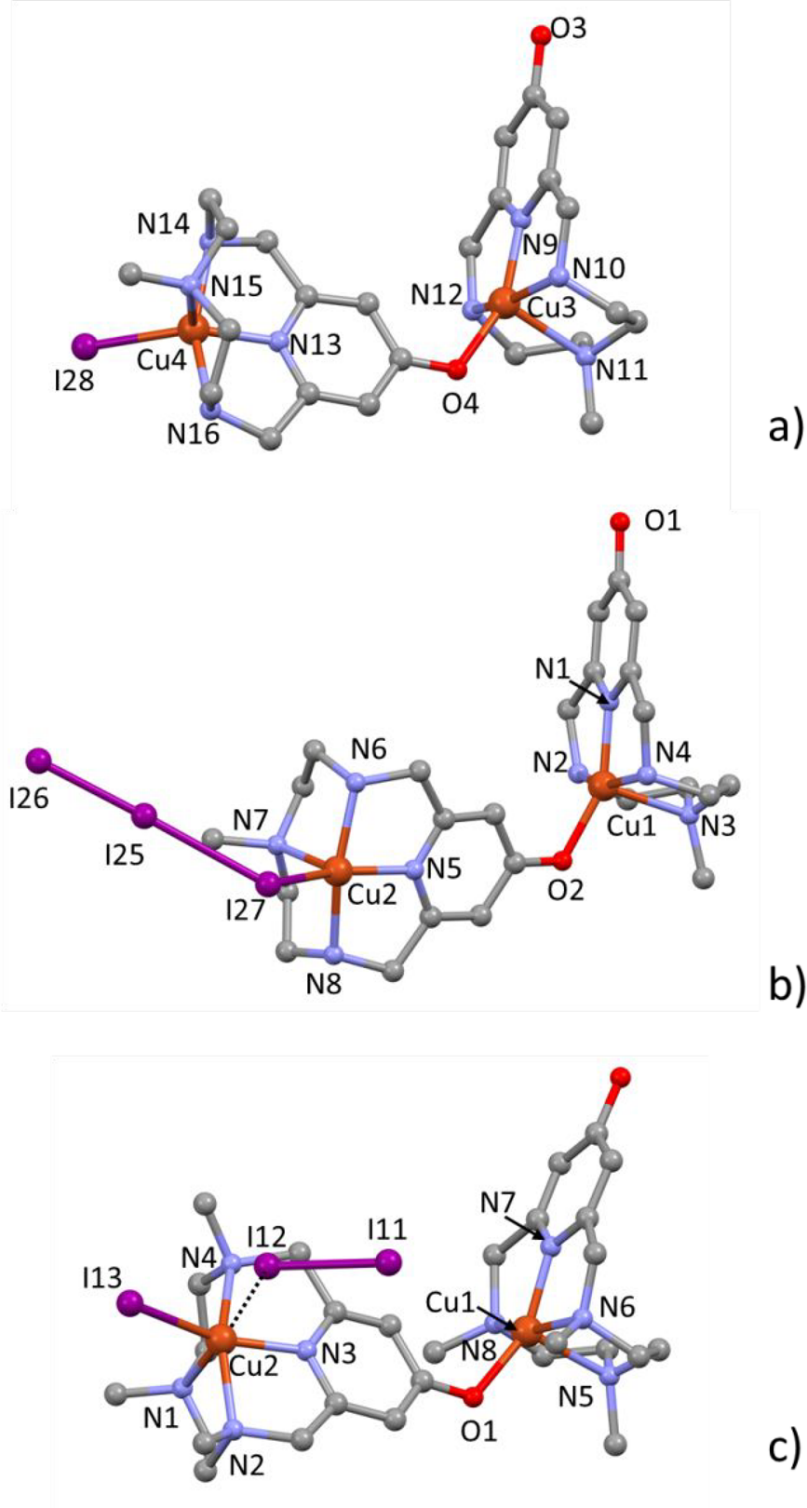
Crystal structures of the [(Cu**L2-Me**)(CuH_–1_**L2-Me**)X]^2+^ dinuclear cations in compound **3**, X = I^–^ (a) or X = I_3_^–^ (b), and of the [(Cu**L2-Me**_**3**_)(CuH_–1_**L2-Me**_**3**_)I]^2+^ dinuclear cations in compound **4** (c); Cu2–I13 2.555(3), Cu2···I12 3.306(4) Å, Cu2···I11 5.324(4) Å.

The resulting formula of the crystalline compounds are {[(Cu**L2-Me**)(CuH_–1_**L2-Me**)I]·[(Cu**L2-Me**)(CuH_–1_**L2-Me**)]I_3_}(I_2_)(I_5_)_3_(I_7_) (**3**) and [(Cu**L2-Me**_**3**_)(CuH_–1_**L2-Me**_**3**_)I](I_2_)_2_(I_5_)_2_ (**4**)). Drawings of the dinuclear cations are shown in Figure 8. Details of coordination geometry (bond distances and angles) are reported in Tables S5 and S6, while bond distances and angles for iodine molecules and polyiodide anions are listed in Tables S7 and S8.

As observed in the previous structures, also in these complexes, the ligands assume the *cis*-folded N_4_ configuration. In each (CuL)^2+^ unit (L = **L2-Me**, **L2-Me**_**3**_), the copper ion is coordinated by the four macrocyclic nitrogen atoms and by the pyridinol oxygen from the (CuH_–1_L)^+^ unit, while in each (CuH_–1_L)^+^ unit, the coordination sphere includes the four macrocyclic nitrogen atoms and an exogenous species which can be a monatomic iodide or a triiodide anion. With only one exception (see description of compound **3**), the coordination geometries are square pyramidal: the nitrogen atom across from the pyridinol group occupies the apical position, while the remaining three nitrogen atoms define the basal plane together with the pyridinol oxygen (in (CuL)^2+^) or the exogenous ligand (in (CuH_–1_L)^+^). In **4**, an iodine atom from an I_2_ molecule occupies, at very long distance (Cu2···I12 3.306(4) Å), the sixth position of a distorted octahedron. A similar copper coordinated I_2_ molecule, featuring Cu–I distance significantly shorter than the sum of the van der Waals radii, was previously reported by Hu et al.^82^ Analogously to compounds **1** and **2**, the two copper units in each [(CuL)(CuH_–1_L)X]^2+^ dinuclear cation are rotated, with respect to each other, with dihedral angles of 81.3° (X = I^–^) and 74.2° (X = I_3_^–^) in **3** and 81.4° in **4** (as measured by the angle between the mean planes of pyridine rings).

**Figure 9:**
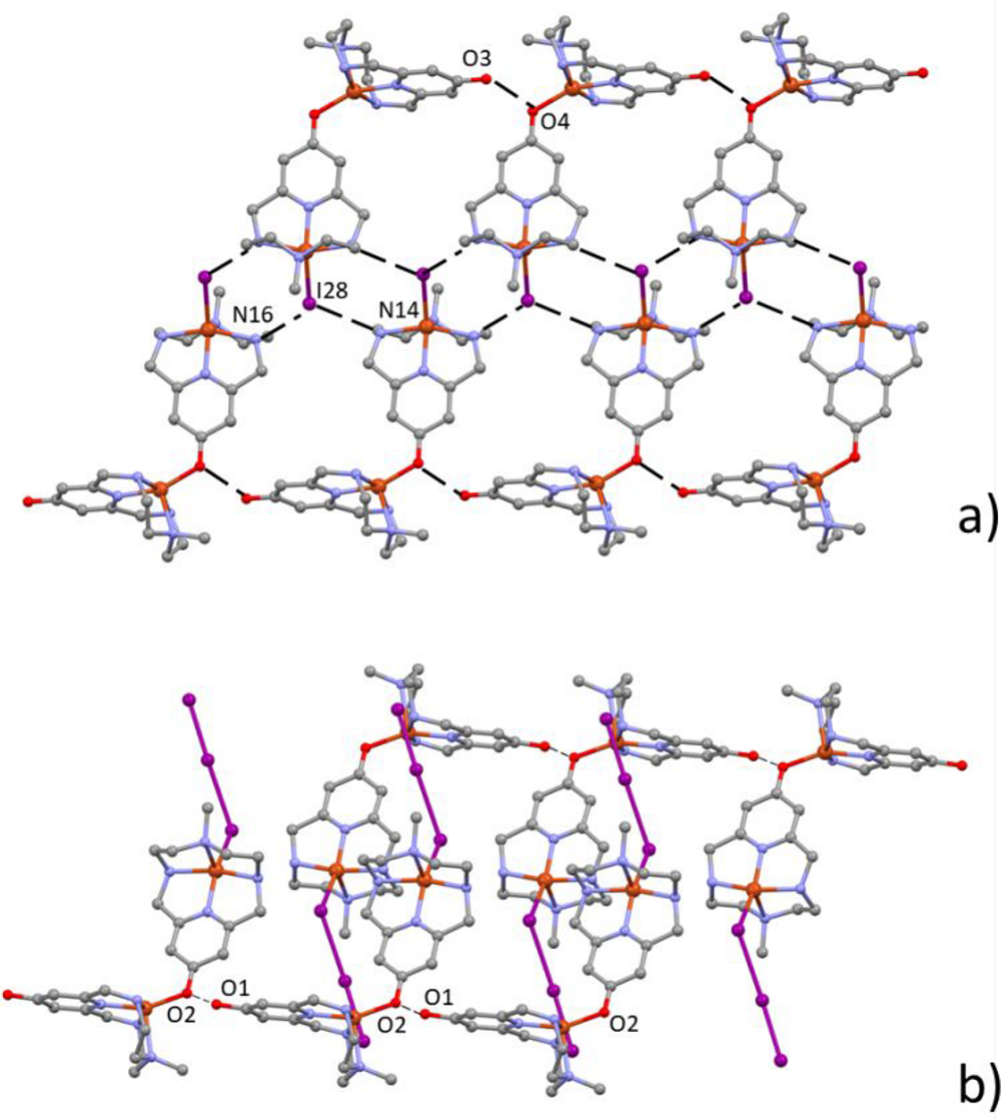
Compound **3**. (a) Array, growing along the *b* axis, of [(Cu**L2-Me**)(CuH_–1_**L2-Me**)I]^2+^ binuclear complexes linked by charge assisted OH···O^–^ and NH···I^–^ H-bonds. (b) Array, growing along the *b* axis, of [(Cu**L2-Me**)(CuH_–1_**L2-Me**)I_3_]^2+^ binuclear complexes linked by charge assisted OH···O^–^ H-bonds.

**Figure 10:**
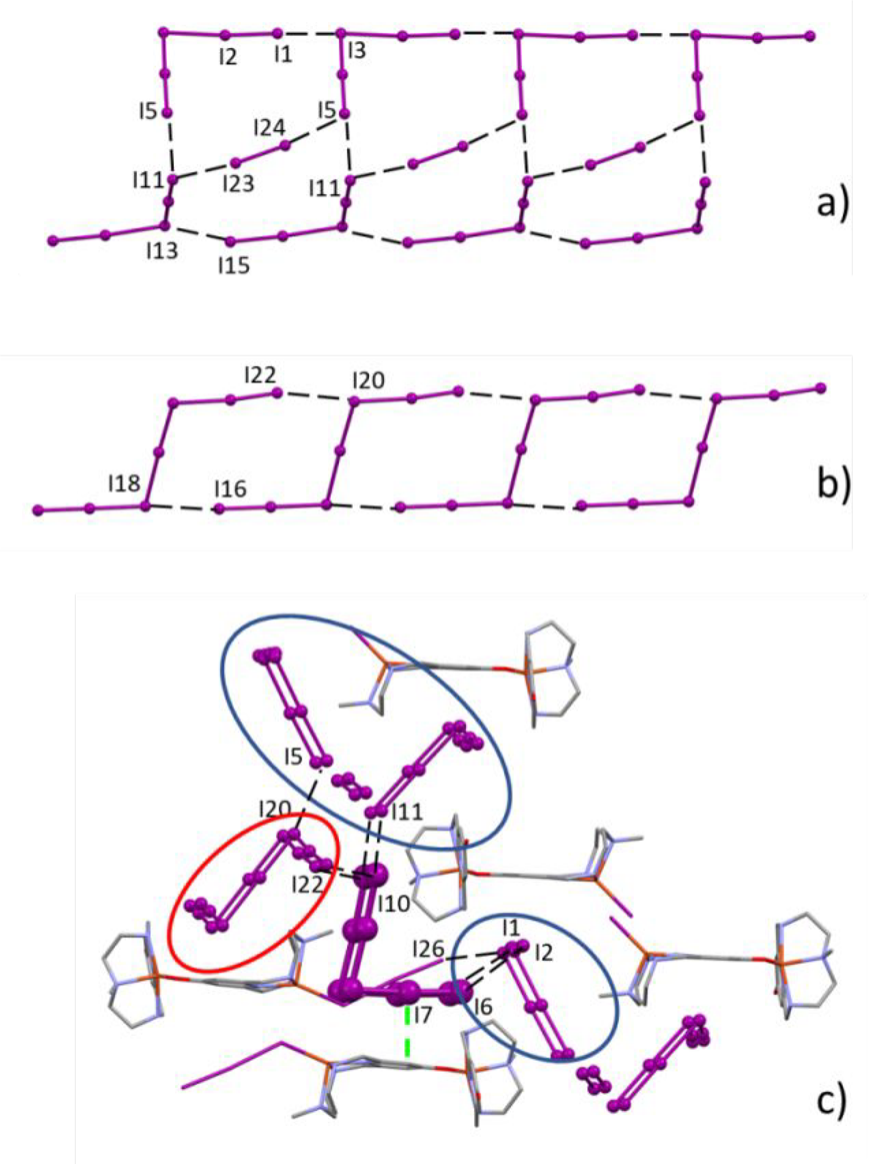
Compound **3**. Tapes of (I_5_^–^ + I_2_)-based 11-atom rings (a) and I_7_^–^-based 10-atom rings (b) joined by secondary I···I bonds. Details for the crystal packing evidencing the pentaiodide anions connecting the I_7_^–^-based 10-atom rings (red circle) and the (I_5_^–^ + I_2_)-based 11-atom rings (blue circles) (c). Anion−π interaction marked by a green dashed line.

The asymmetric unit of **3** contains two [(Cu**L2-Me**)(CuH_–1_**L2-Me**)X]^2+^ binuclear complexes which differ in X, this being I^–^ in one case and an end-on coordinated I_3_^–^ anion in the other (Figure 8a,b). The I^–^ is disordered and spread over two positions, which account for the 65% (I28) and the 35% (I29) of the overall iodide electron density. All figures, tables, and discussions reported here refer only to the most populated I28 (see Figure S8 for the I29 minor component). The coordination sphere of the (CuH_–1_**L2-Me**)I unit (Figure 8a), which represents the exception mentioned above, can be best described as a distorted trigonal bipyramid having the iodide, the pyridine N13 and the methylated N15 nitrogen atoms in the equatorial plane, and the two benzylic nitrogen atoms in the apical positions. Both [(Cu**L2-Me**)(CuH_–1_**L2-Me**)X]^2+^ (X = I^–^, I_3_^–^) interact with their own copies translated along the *b* axis (Figure 9), giving rise to rather strong^83^ charge assisted OH···O^–^ H-bonds involving their protonated and deprotonated pyridinol oxygen atoms (2.59(1) Å for O1···O2^–^ in [(Cu**L2-Me**)(CuH_–1_**L2-Me**)I_3_]^2+^ and 2.60(1) Å for O3···O4^–^ in [(Cu**L2-Me**)(CuH_–1_**L2-Me**)I]^2+^). In [(Cu**L2-Me**)(CuH_–1_**L2-Me**)I]^2+^, adjacent cations form additional NH···I^–^ H-bonds with the coordinated I28 anions (Figure 9a, N16···I28, 3.53(1) Å; N14···I28, 3.61(1) Å). By this way, chains of equivalent binuclear complexes develop along the *b* axis, which are completely surrounded by an intricate network of polyiodide anions and iodine molecules. Several I···I contacts are very short and can be described as secondary bonds, being below the accepted threshold of 3.7 Å.^37^ Some of them (I1···I3, 3.388(2) Å; I11···I23, 3.384(1) Å) are just above the boundary between intra- and intermolecular distances (3.3 Å).^84^ These networks are shown in Figure 10, while Table 4 lists relevant I···I contacts. The chains formed by the [(Cu**L2-Me**)(CuH_–1_**L2-Me**)I]^2+^ cations are surrounded by polyiodide anions and iodine molecules arranged in ribbons of 11-atom rings (Figure 10a), while in the case of [(Cu**L2-Me**)(CuH_–1_**L2-Me**)I_3_]^2+^ chains, the array of metal complexes is surrounded by I_7_^–^ anions which generate tapes of 10-atom rings (Figure 10b).

**Table 4:** Selected Contacts (Å) Involving Iodine Atoms in Compound **3**

N14···I28	3.61(1)	I15···I13	3.733(2)
N16···I28	3.53(1)	I22···I10	3.755(1)
I1···I3	3.388(2)	I11···I5	3.798(1)
I11···I23	3.384(1)	I1···I26	3.821(2)
I5···I24	3.500(2)	I2···I6	3.850(2)
I10···I11	3.567(1)	I16···I18	3.886(1)
		I5···I20	3.935(2)

Analyses of the shortest I···I contacts in the crystal packing evidence the role of an additional pentaiodide anion, which connects the I_7_^–^-based 10-atom rings with the 11-atom rings based on I_5_^–^ + I_2_. Notably, the latter 11-atom rings are also in direct contact with the coordinated triiodide of [(Cu**L2-Me**)(CuH_–1_**L2-Me**)I_3_]^2+^ (I1···I26, 3.821(2) Å) (Figure 10c).

This gluing pentaiodide anion gives a very short anion-π contact with the pyridinol ring in (CuH_–1_**L2-Me**)]I_3_ (I7···ring centroid, 3.6 Å; Figure 10c). A strong anion−π interaction also involves the pyridinol ring of (CuH_–1_**L2-Me**)I and one of its surrounding pentaiodide (I3···ring centroid, 3.6 Å; Figure S9). As shown below, the involvement of the pyridinol group in two coordination bonds (through the pyridine nitrogen and the deprotonated pyridinol oxygen), established by both (CuH_–1_**L2-Me**)I_3_ and (CuH_–1_**L2-Me**)I units, greatly depletes the aromatic ring of its electron density, so providing an important and peculiar anchor point for polyiodide binding.

**Figure 11:**
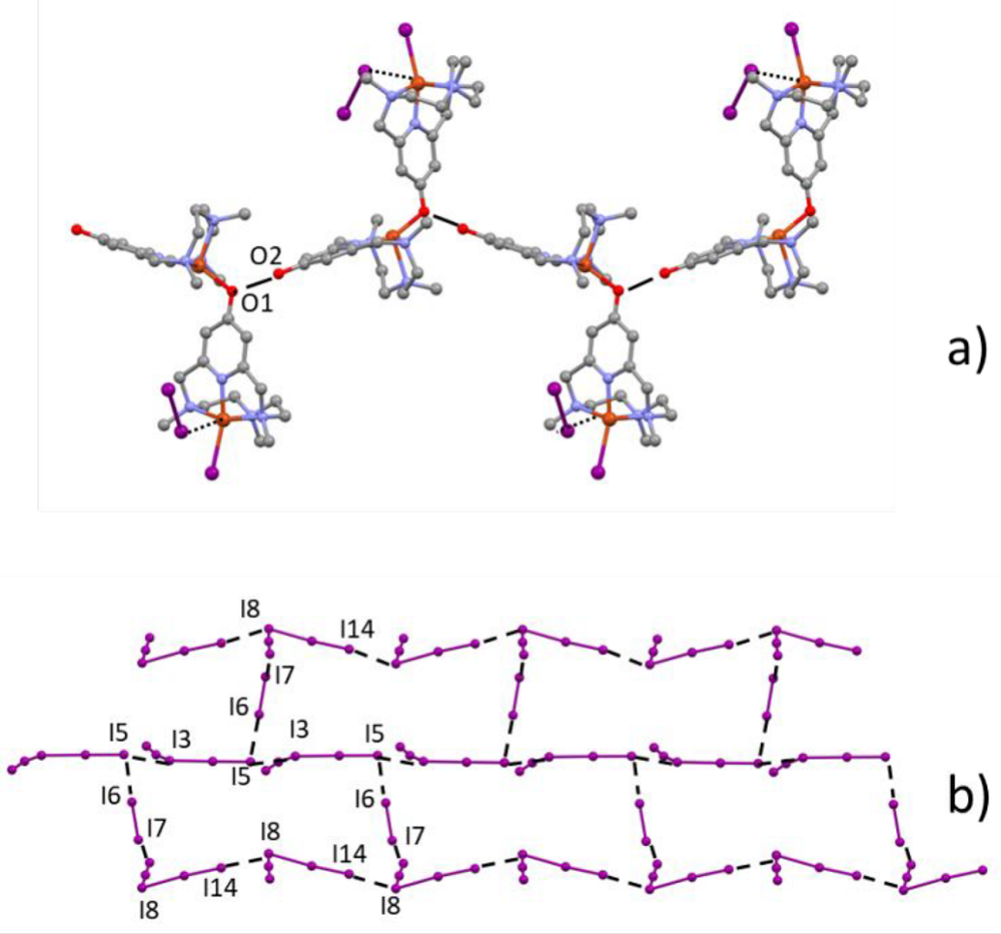
Compound **4**. Array, growing along the *b* axis, of [(LCu)(LH_–1_Cu)I]^2+^ binuclear complexes linked by charge assisted OH···O^–^ H-bonds. Iodine molecules, occupying the sixth coordination position of a distorted octahedron around Cu1, are also shown (a). 18-membered rings based on iodine molecules and pentaiodide anions (b).

The crystal structure of [(Cu**L2-Me**_**3**_)(CuH_–1_**L2-Me**_**3**_)I](I_2_)_2_(I_5_)_2_ (**4**) shows strong similarity with the structures of compound **3**. Despite the presence of an iodine molecule occupying the sixth position of a distorted octahedron (Figure 8c), the [(Cu**L2-Me**_**3**_)(CuH_–1_**L2-Me**_**3**_)I]^2+^ dinuclear cations form chains developing along the *b* axis, where protonated and deprotonated pyridinol oxygen atoms from neighboring units give strong OH···O^–^ H-bonds (Figure 11a).

The remaining not coordinated iodine molecules and the pentaiodide anions form I···I secondary bonds which define the 2D grid shown in Figure 11b (selected I···I distances are listed in Table 5). The grid meshes, constituted by 18-atom rings, are in contact with the coordinated iodine molecules giving iodine–iodine contacts which are well below the iodine–iodine secondary bond threshold (I3···I11 and I12···I13 distances 3.476(3) and 3.361(3) Å, respectively, Figure S10). A few longer I···I contacts contribute to the overall stabilization of the crystal packing (Table 5 and Figure S10).

**Table 5:** Selected Contacts (Å) Involving Iodine Atoms in Compound **4**

I3···I5	3.365(3)	I8···I14	3.560(2)
I12···I13	3.361(3)	I1···I7	3.738(3)
I5···I6	3.430(2)	I1···I8	3.945(2)
I7···I8	3.466(2)	I11···I10	3.938(3)
I3···I11	3.476(3)		

### Considerations on the Crystal Structures: Hirshfeld Surface Analysis and I···I Contacts Details

Percentage composition of Hirshfeld surfaces,^63−65^ in terms of atoms in contact, is found very similar among the units [(Cu**L2-Me**)(CuH_–1_**L2-Me**)I]^2+^, formally coordinated by I_3_^–^, [(Cu**L2-Me**)(CuH_–1_**L2-Me**)I]^2+^, formally coordinated by I^–^, and [(Cu**L2-Me**_**3**_)(CuH_–1_**L2-Me**_**3**_)I]^2+^ (Figure S11, Table S9), as expected for similar molecules surrounded by polyiodides networks.

**Figure 12:**
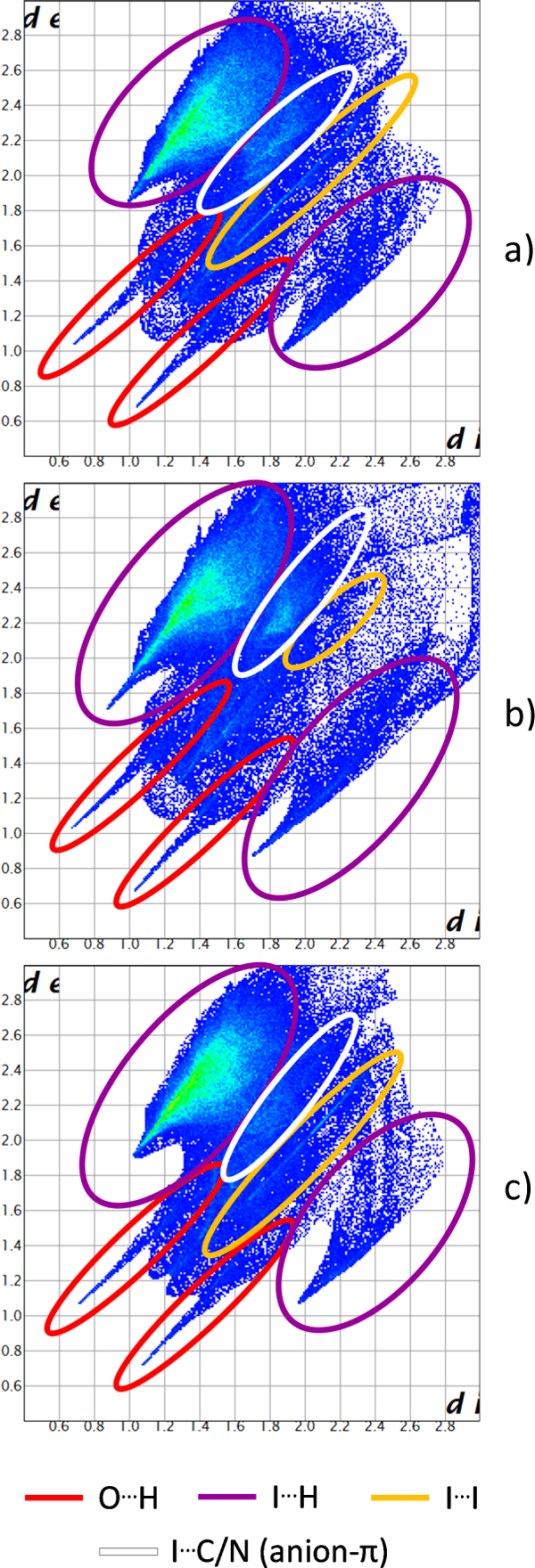
Global fingerprint plots for: **3**, surface of [(CuL)(CuH_–1_ L)I]^2+^ formally coordinated by I_3_^–^ (a); **3**, surface of [(CuL)(CuH_–1_ L)I]^2+^ formally coordinated by I^–^ (b); **4**, surface of [(CuL)(CuH_–1_ L)I]^2+^ (c). For plots related to individual types of contact, see Figure S11.

Far more insights can be obtained from comparison of the related fingerprint plots (Figure 12). The most prominent shared feature among fingerprint plots of these **L2** derivatives is the appearance of two O···H tips typical of strong and directional H-bonding (Figure 12, red circles). Of course, O···H contacts were absent in **L1**-based structures (they do not have an −OH function). On the contrary, H···I type contacts (Figure 12, purple circles) pass relatively unmodified from **L1**- to **L2**-based structures.

**Figure 13:**
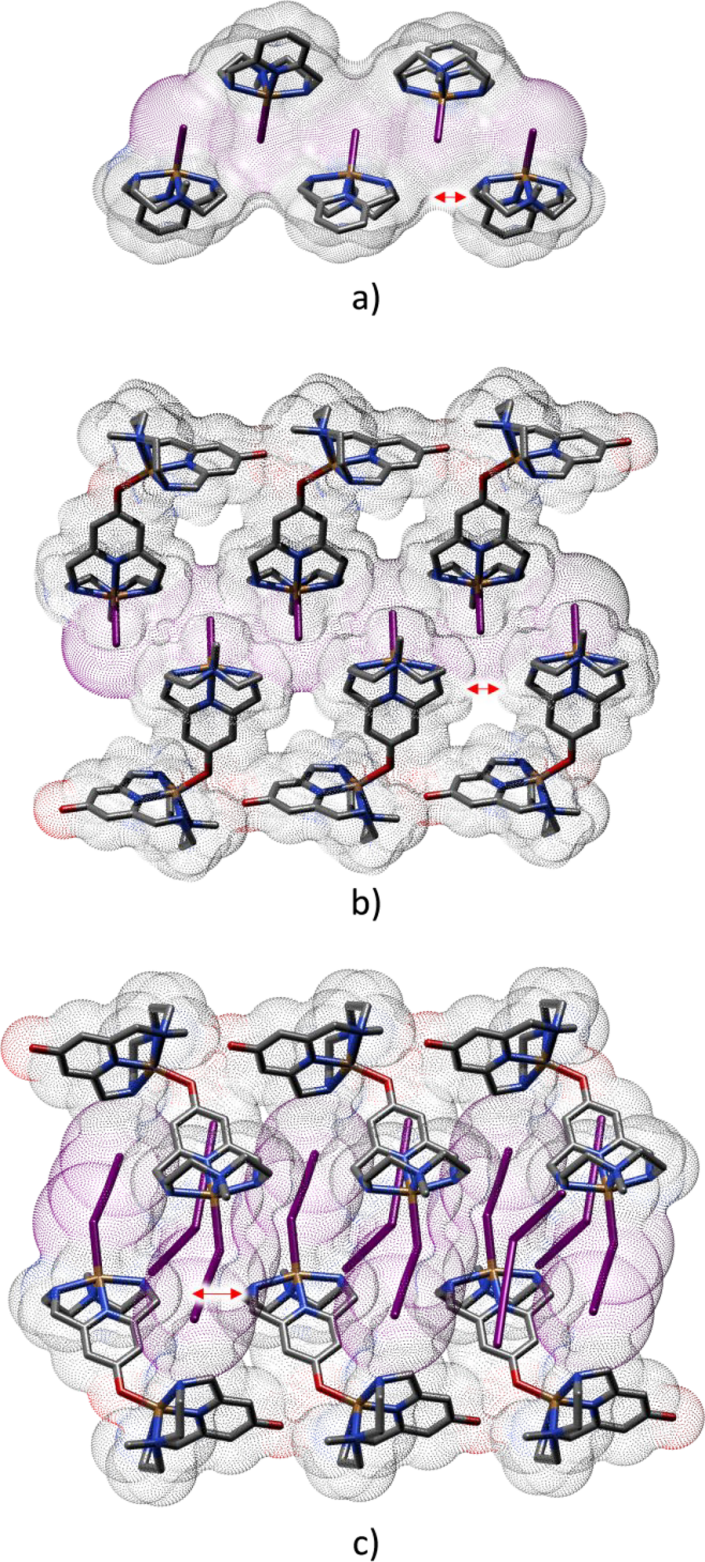
NH···I H-bonded ribbons as found in **L1-Me**-based crystals^30^ (a) and in **3** (coordinated I^–^, (b) and I_3_^–^, (c), respectively).

In the case of **L-Me** (**L** = **L1**, **L2**), the [(Cu**L-Me**)I]^+^ tectons organize themselves in ribbons held together by long NH···I interactions involving the coordinated iodide anion (Figures 9 and 13). The subtending network adapts from the relatively unconstrained situation found in the **L1-Me** complex (shortest NH···I distances, Figure 13a), to the more hindered situation of **L2-Me** (Figure 13b), where additional space is required to accommodate the dinuclear complex, to the crowded situation generated by the presence of coordinated I_3_^–^ (Figure 13c), where making room for the protruding I_3_^–^ requires involvement of further polyiodides (I_5_^–^) to keep the chain in place, thus increasing the spacing within complexes in the ribbon. As a matter of fact, the two types of H-bond donor–acceptor pairs, that is, NH···I and OH···O, do never mix up.

C···I and N···I contacts (Figure 12, white circles), mostly related to anion-π type interactions,^28,30,85,86^ are significantly more developed for **L2**-based structures than for **L1**-based ones. Given the pivotal role of ring electrostatic potential/polarization,^28,87−91^ the more relevant role of these interactions is most likely due to the superior polarization of the aromatic portion of **L2** derivatives, especially when both amino and phenolic functions coordinate to Cu(II).

**Figure 14:**
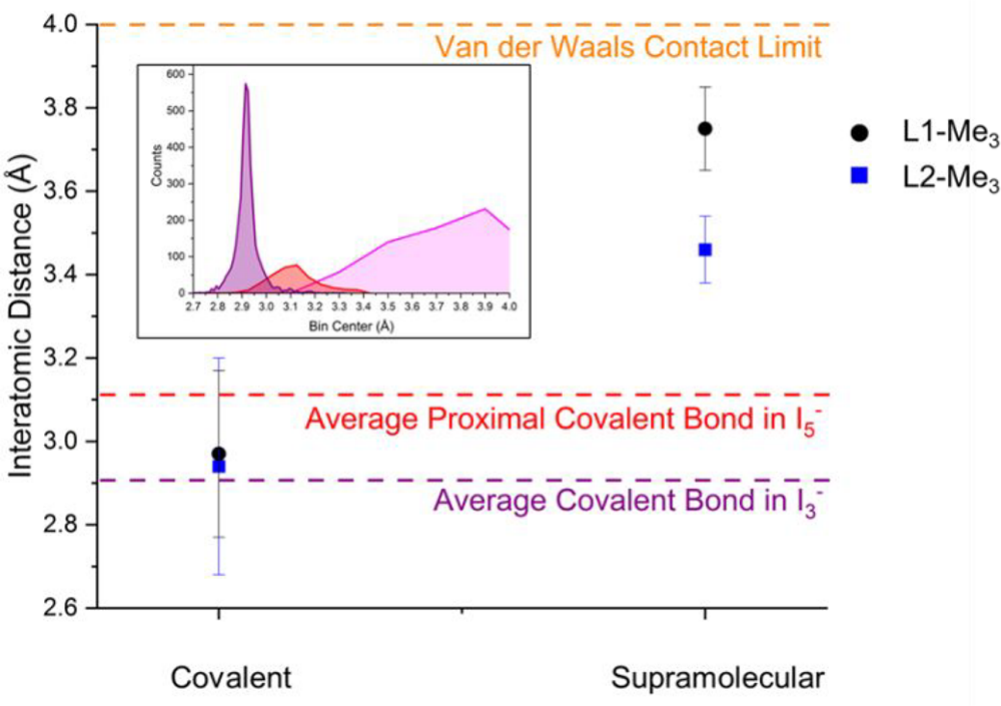
Comparison of I–I covalent and supramolecular distances as found in **L1-Me**_**3**_- and **L2-Me**_**3**_-containing crystalline phases (average value, standard deviation as error bar). Relevant reference distances (average I–I bond length in I_3_^–^, proximal covalent bond in I_5_^–^ and van der Waals contact distance) are also reported to scale for comparison purposes. Inset shows (left to right) covalent bond distribution for I_3_^–^ (violet) and interaction distances of I_3_^–^ subunits with generic I_2_ probes, either covalent (i.e., pentaiodide proximal bond distances, red) or supramolecular (pink), in order to give an appropriate view of actual broadness of statistical distributions. Reproduced with permission from ref (36). Copyright 2021 Royal Society of Chemistry.

Coming to polyiodides and I···I contacts (Figure 12, yellow circles, and Figure S12d), it is relatively clear—notice the tip that shows the mutual piercing of interacting partners into one another electronic density—that the difference between Cu–I···I_2_ in **3** (Figure S12d, left column), which we interpreted as a Cu-bound I_3_^–^, and I^–^···I_2_ distance in Cu(II) coordination sphere in **4** (Figure S12d, right column), which we interpreted as individual I^–^···I_2_ entities, is rather tiny, while it is enormous with respect to polyiodides merely touching themselves (Figure S12d, central column). A real discrimination between fully covalent and fully supramolecular contacts can hardly happen in the spatial region that goes from 3.1 to 3.4 Å (Figure 14). As discussed elsewhere,^36,37^ variability of I–I covalent bonds is already large for triiodides. A Lorentzian curve centered at 2.9179(1) Å and possessing a full width at half-maximum of 0.0436(3) Å (*R*^2^ = 0.997, χ^2^ = 13.27) best describes I_3_^–^ experimental bond lengths. Accordingly, bond lengths ≥3.1 Å are already quite odd for triiodide, as they already fall well above the center + 3 fwhm level. The “Gaussian” tends to get smeared further already for pentaiodides (for I_5_^–^ mean distal I–I bond length ±1 standard deviation is 2.80 ± 0.04 Å, while the proximal one is 3.1 ± 0.1 Å, Figure 14).

Cu(II)-coordinated triiodide in **3** and Cu(II)-coordinated I_2_ in **4** are clearly borderline cases. In the latter, the I_2_ molecule is virtually equidistant from Cu(II) (3.305 Å) and coordinated I^–^ (3.361 Å) (i.e., with respect to sum of the ionic or van der Waals radii, I···I interaction should be stronger than I···Cu), being coordinated at rather unusual angles with respect to both the metal center (I–I–Cu angle of 122.7°, i.e. significantly larger than tetrahedral) and the coordinated I^–^ (I–I···I^–^ angle of 165.9°, i.e. significantly smaller than 180°).

We commented above the similarity of Cu(II) complexes with **L1-Me** and **L2-Me** in giving H-bonded ribbons. It is now interesting to extend such comparison to polyiodide complexes produced by Cu(II) complexes of **L1-Me**_**3**_ and **L2-Me**_**3**_. As observed before, complete removal of H-bond donors by methylation led to crystal structures whose directionality is dominated by I···I interactions. In the case of [Cu(**L1-Me**_**3**_)I]^+^, this led to strong interaction with an I_2_ molecule (assigned as coordinated I_3_^–^, Cu–I···I_2_ distance of 3.244 Å, same distance is 3.237 in **3**, cf. above discussion) and catenation with pentaiodides in forming 11-membered polyiodide rings fused in a chain. The covalent (2.97 ± 0.21 Å) and supramolecular (3.75 ± 0.01 Å) nature of the interactions within said rings remained quite manifest from interatomic distances (Figure 14).

The situation in **4** is hardly comparable, most likely because of the constraints imposed by OH···O interactions (apparently indifferent to the degree of N-methylation) and bulkiness of the dinuclear complexes. Notwithstanding such intrinsic differences, an 18-atom tile (Figure 11b) is clearly distinguishable as the main repeating pattern. Within such a grid, we observe that average length of covalent bonds is not significantly different from above **L1-Me**_**3**_ data, but the distinction between covalent bonds (in **4**, 2.94 ± 0.26) and supramolecular contacts (in **4**, 3.46 ± 0.08) is now significantly reduced (Figure 14).

This observation cannot be *tout court* ascribed to iodine density, which is slightly lower for **4** than for [Cu(**L1-Me**_**3**_)(I·I_2_)](I_5_)^30^ (I_*N*_ = 0.443 for **4** vs 0.472 for the latter),^49^ nor it is easily justified by simply counting charges per ring, seeking to prove a lessened electrostatic repulsion among polyiodides: In order to do so, one should postulate to know where charges are localized in the first place. This is in direct conflict with the (sounder) idea that intraring supramolecular bond lengths in **4** are significantly shortened because of partial charge transfer. If such is indeed the case, we can conclude that with **L2-Me**_**3**_, relative to **L1-Me**_**3**_, we come much closer to a real polyiodide network that can be hardly considered as discrete anions due to their strong mutual interactions.

### Electronic Spectra

The UV–vis spectrum of the Cu(II) complex with **L2**, obtained in aqueous solution at pH 6, had already been reported.^48^ It consists of an intense π → π* charge-transfer band at 249 nm with a shoulder in the 284–330 nm region, typical of the ligand, and a very weak and broad d–d band around 699 nm. We have now recorded the spectra of the Cu(II) complexes with the three pyridinol ligands considered here, in the 200–800 nm region and under pH conditions corresponding to about 100% formation of the (CuH_–1_L)^+^ (L = **L2**, **L2-M2**, **L2-Me**_**3**_) species. In the UV region (Figure S13), all three complexes show charge-transfer bands at 248–251 nm, with a shoulder in the 260–275 nm region, which are practically coincident, though somewhat more intense and slightly blue-shifted, with those recorded for the uncomplexed ligands in the same pH conditions (Figure S14). Another smoother and weaker band is clearly visible at about 305 nm in the spectrum of (CuH_–1_**L2-Me**_**3**_)^+^ that becomes less evident in the spectra of the other two complexes. Very strong ligand-centered transitions (not characterized) are present below 220 nm (Figure S13).

The visible region of these spectra is characterized by broad and weak d–d bands centered around 680 nm [(CuH_–1_**L2**)^+^], 682 nm [(CuH_–1_**L2-Me**)^+^], and 656 nm [(CuH_–1_**L2-Me**_**3**_)^+^] that are responsible for the blue color of the complexes (Figure S13).

In the electronic spectra of the solid polyiodide complexes **3** and **4**, the bands of the complexes below 400 nm overlap the bands of the polyiodide anions. The strong ligand-centered transition below 220 nm is still present, while two bands at 301/305 nm and 400/411 nm characterize the spectra of **4**/**3** (Figure S15). These bands are consistent with the presence of I_2*n*+1_^–^ (*n* = 1–3) anions and are located at relatively low energies, especially the 400/411 nm ones, in agreement with the content of I_5_^–^ and I_7_^–^ anions.^37^ Tails of the 400/411 nm bands extend toward longer wavelengths in accordance with the presence of lower energy absorption components expected for polyiodides higher than I_3_^–^, which are more clearly evidenced by the shoulder emerging at about 600 nm in the spectrum of **4**.

## Conclusions

As expected, the presence of the ionizable hydroxyl group on the pyridine ring led to the involvement of the pyridinol group in the coordination to two metal ions. This does not occur in solution, where only Cu(II) complexes with 1:1 metal:ligand stoichiometry are formed, while, in the solid state, bridging coordination of the hydroxyl group gives rise to polymeric coordination chains of general formula {[Cu(H_–1_L)]}_*n*_^*n*+^ (L = **L2-Me**, **L2-Me**_**3**_). The presence in solution of the I^–^/I_2_ couple induces crystallization of compounds in which this polymerization tendency stops with the formation of [(CuL)(CuH_–1_L)]^3+^ (L = **L2-Me**, **L2-Me**_**3**_) dimers that are surrounded by polyiodide networks.

While these new features become immediately evident in the comparison between **L1**- and **L2**-based ligands, the understanding of how these structural changes correlate with the formation of different polyiodide networks appears to be challenging, especially because of the large structural difference between the involved cations, which are monomeric complexes, in the case of **L1**-based ligands, and dimeric complexes in the case of the **L2**-based analogues. Nevertheless, this challenging task is central to the main goal of this work, that is, the understanding of how covalent, coordination, and supramolecular forces can be used, mixed together, to construct robust polyiodide networks extending across the crystals, bearing in mind the applicative opportunity of using these compounds as solid-state conductors.

First of all, we can highlight a common feature of the two series of ligands: For both of them, the formation of polyiodide networks appears to be dominated by H-bonds until full ligand N-methylation is achieved, then, I···I interactions become the main directional forces. That is, N-methylation can be helpfully used to gradually shift the directional control of the assembly process from H-bonds, mainly involving complex units, to I···I interactions, mainly involving iodine-based units.

Nevertheless, hydrogen bonding by the pyridinol −OH group, which is indifferent to the degree of N-methylation of the ligands, determines the formation of complex chains functioning as molds for the construction of polyiodide ribbons (constituted by 10- and 11-atom rings in **3** and by 18-atom rings in **4**) that run parallel to the complex chains and interact with them via I···I interactions, involving metal-coordinated iodine atoms (I_3_^–^ in **3** and I_2_ in **4**), and short anion−π contacts.

**Figure 15:**
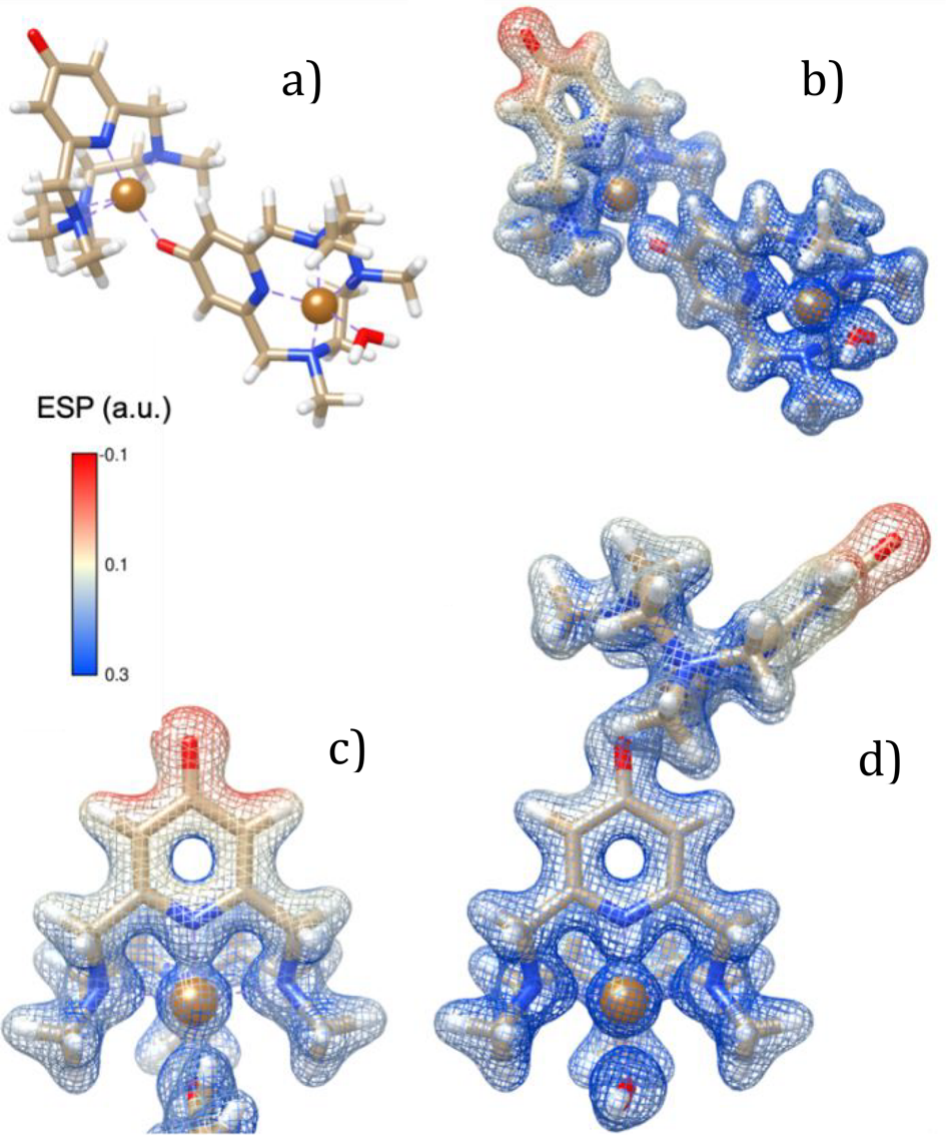
Representation of the ESP maps of [(Cu**L2-Me**_**3**_)(CuH_–1_**L2-Me**_**3**_)I]^2+^. (a) Representation of the dimeric complex. (b) Representation of the ESP map of the dimeric complex, overlaid to the representation of its structure. (c) Detail of the ESP map of the pyridinol ring in which the oxygen atom is not interacting with a copper atom. (d) Detail of the ESP map of the pyridinol ring in which the oxygen atom is bonded to the copper atom of the second complex.

With respect to the latter, anion−π interactions appear to be significantly more developed for **L2**-based structures than for **L1**-based ones, thanks to the deprotonated pyridinol groups involved in O- and N-coordination to Cu(II) ions. It is known that azines may act as π-acid ligands, which can bind anions above their centers according to their positive ESPs and their molecular quadrupole moments.^28^ Indeed, *s*-tetrazine, the most polarized molecule of this family (Q_*zz*_ quadrupole moment of 11.4 Buckinghams), is prodigal in forming anion−π interactions,^89^ even in solution, as recently shown by some of us.^89,92−97^ Nonetheless, also pyridine, despite being the less polarized azine (Q_*zz*_ quadrupole moment of 3.0 Buckinghams), forms anion−π complexes,^28^ especially when they are activated by electron-withdrawing substituents^98^ or by N-interaction with positive charges (H^+^, metal ions).^99,100^ Insertion of an electron-donating −OH group on the pyridine rings is expected to hinder anion−π interactions, but deprotonation of this hydroxyl function and coordination to metal ions of both O and N donors of the pyridinol group, like in our complexes, turns the negatively charged surface of the ring into a markedly positive surface (Figure 15) capable of attracting anions.

Finally, it is worth noting that supramolecular I···I interactions are shorter in **L2**-based polyiodides, relative to **L1**-based ones, despite the fact that the iodine density (iodine number I_*N*_ = 0.454 for **3** and I_*N*_ = 0.443 for **4**) does not follow the same trend, due to the bulkier dimeric complex cations. Especially in the case of **4**, such shortening of the I···I contacts considerably blurs the identity of the individual iodine-based components in favor of iodine networks in which the orbital overlap, essential for the Grotthuss-type conduction mechanism,^37,38^ gains importance.

## Supporting Information Available

Distribution diagrams, tables of crystallographic bond distances and angles, Hirshfeld surface compositions and plots, crystal data and refinement, additional views of the crystal structures, ^1^H NMR and UV–vis spectra (PDF)

## Accession Codes

CCDC 2062421–2062422 and 2094997–2094998 contain the supplementary crystallographic data for this paper. These data can be obtained free of charge via www.ccdc.cam.ac.uk/data_request/cif, or by emailing data_request@ccdc.cam.ac.uk, or by contacting The Cambridge Crystallographic Data Centre, 12 Union Road, Cambridge CB2 1EZ, UK; fax: +44 1223 336033.
