# Supplementary material for: Assembly of Polyiodide Networks with Cu(II) Complexes of Pyridinol-Based Tetraaza Macrocycles
Source: Inorg Chem. 2021 Dec 22;61(1):368–83. doi: 10.1021/acs.inorgchem.1c02967 (PMC8753606; doi:10.1021/acs.inorgchem.1c02967)
Supplement: Supplementary file 1 — ic1c02967_si_001.pdf [file ic1c02967_si_001.pdf]

Supporting Information

Assembly of polyiodide networks with Cu(II) complexes of pyridinol-based tetraaza macrocycles.

Álvaro Martínez-Camarena,[a] Matteo Savastano,[b] Salvador Blasco,[a] Estefanía Delgado-Pinar,[a,c] Claudia Giorgi,[b] Antonio Bianchi,[*b] Enrique García-España[*a] and Carla Bazzicalupi[b]

[a] ICMol, Department of Inorganic Chemistry, University of Valencia, C/Catedrático José Beltrán 2, Paterna, Spain. E-mail: enrique.garcia-es@uv.es.

[b] Department of Chemistry "Ugo Schiff", University of Florence, Via della Lastruccia 3-13, 50019 Sesto Fiorentino, Italy. E-mail: antonio.bianchi@unifi.it.

[c] Department of Chemistry, CQC, University of Coimbra, P3004-535 Coimbra, Portugal.

Content

Table S1. Crystal data and refinement parameters for **1**-**4**. S3
T**able S2.** Logarithms of stepwise protonation constants of **L2-Me** obtained by ^{1}H NMR measurements and potentiometric measurements (pot.). S4
Table S3: Bond distances (Å) and angles (°) for the crystal structure of [Cu(H_{-1}**L2-Me**)](ClO_4) (**1**). S4
Table S4: Bond distances (Å) and angles (°) for the crystal structure of [Cu(H_{-1}**L2-Me₃**)](ClO_4) (**2**). S5
Table S5: Bond distances (Å) and angles (°) for the crystal structure of {[(Cu**L2-Me**)(CuH_{-1}**L2-Me**)I]·[(Cu**L2-Me**)(CuH_{-1}**L2-Me**)]I_3}(I_2)(I_5)$_3$(I_7) (**3**). S6
Table S6: Bond distances (Å) and angles (°) for the crystal structure of [(Cu**L2-Me₃**)(CuH_{-1}**L2-Me₃**)I](I_2)$_2$(I_5)$_2$ (**4**). S7
Table S7. Bond distances (Å) and angles (°) for iodine molecules and polyiodide anions in compound **3**. S8
Table S8. Bond distances (Å) and angles (°) for iodine molecules and polyiodide anions in compound **4**. S9
Table S9. Percentual breakdown of Hirshfeld surface contribution by element for cases illustrated in Fig. 16. .... S10
Fig. S1. Distribution diagrams of the protonated species formed by **L2-Me** and **L2-Me₃** as a function of pH in aqueous solution and pH-dependent UV-Vis spectral data. S11
Fig. S2. Overlay ^{1}H-NMR spectra of **L2-Me** measured in D_2O solution from pD 0.80 to 11.83.. S12
Fig. S3. Overlay of the ^{1}H-NMR spectra of **L2-Me** measured in D_2O solution from pD 1.18 to 13.02. .... S13
Fig. S4. Derivatives of calculated chemical shifts and of a protonation site occupancy (theta) with respect to pD. S14
Fig. S5. Distribution diagrams of the complexes formed in the systems Cu(II)/**L2-Me** and Cu(II)/**L2-Me₃**. S15
Fig. S6. Helical arrangement of the coordination polymers in [Cu(H_{-1}**L2-Me**)](ClO_4)·0.716H_2O (**1**) and [Cu(H_{-1}**L2-Me₃**)](ClO_4)·H_2O (**2**). S16
Fig. S7. View of a pair of polymeric chains found in **1**. S17
Fig. S8. Array, growing along the b axis, of [(Cu**L2-Me**)(CuH_{-1}**L2-Me**)I]$^{2+}$ binuclear complexes linked by charge assisted OH···O^- and NH···I^- H-bonds in the I29 minor component. S18
Fig. S9. Compound **3.** Array, growing along the b axis, of [(Cu**L2-Me**)(CuH_{-1}**L2-Me**)I]$^{2+}$ binuclear complexes surrounded by tapes of triiodide anions and iodine molecules. Anion-π interaction also involving the pyridinol ring of (CuH_{-1}**L2-Me**)I. S19
Fig. S10. Compound **4**. Contacts established between the grids' pentaiodide atoms and the iodine molecules weakly interacting with Cu1. .... S20
Fig. S11. Overview of BB Hirschfeld surface composition for polyiodide crystal structures. S21
Fig. S12. Fingerprint plots features for Hirshfeld surfaces of **3** and **4**. S22
Fig. S13. Uv-vis spectra of [CuH_{-1}**L2**]$^+$, [CuH_{-1}**L2-Me**]$^+$ and [CuH_{-1}**L2-Me₃**]$^+$. S23
Fig. S14. Uv-vis spectra of **L2**, **L2-Me** and **L2-Me₃**. S24
Fig. S15. UV-vis spectra of thin films of **3** and **4**. S25

Table S1. Crystal data and refinement parameters for [Cu(H$_{-1}$**L2-Me**)](ClO$_4$) (**1**), [Cu(H$_{-1}$**L2-Me$_3$**)](ClO$_4$) (**2**), {[(Cu**L2-Me**)(CuH$_{-1}$**L2-Me**)I]·[(Cu**L2-Me**)(CuH$_{-1}$**L2-Me**)]I$_3$}(I$_2$)(I$_5$)$_3$(I$_7$) (**3**) and [(Cu**L2-Me$_3$**)(CuH$_{-1}$**L2-Me$_3$**)I](I$_2$)$_2$(I$_5$)$_2$ (**4**).

	**1**	**2**	**3**	**4**
Empirical formula	$C_{24}H_{40.86}Cl_2Cu_2N_8O_{11.43}$	$C_{28}H_{48}Cl_2Cu_2N_8O_{11}$	$C_{48}H_{78}Cu_4I_{28}N_{16}O_4$	$C_{28}H_{47}Cu_2I_{15}N_8O_2$
Formula weight	822.39	870.72	4750.62	2558.31
Temperature (K)	104	120	100	100
space group	*C 2/c*	*P 2$_1$/n*	*P2$_1$/c*	*P2$_1$*
a (Å)	15.6485(7)	9.1773(3)	31.338(7)	12.721(2)
b (Å)	17.4067(7)	15.7536(5)	9.362(2)	18.111(2)
c (Å)	23.5708(8)	12.7820(5)	38.502(6)	12.820(2)
β (°)	91.423(2)	103.290(1)	111.443(5)	102.331(6)
Volume (Å^3)	6418.4(4)	1798.5(1)	10514(4)	2885.3(6)
Z	8	2	4	2
Independent reflections / R(int)	16416/0.0413	9620/0.0796	17929/0.1263	11794/0.1041
μ (mm^{-1})	1.564 (Mo-Kα)	1.399 (Mo-Kα)	9.063 (Mo-Kα)	8.795 (Mo-Kα)
R indices [I>2σ(I)]*	R1=0.0304<br>wR2=0.0760	R1=0.0619<br>wR2=0.1559	R1 = 0.0458<br>wR2 = 0.0963	R1 = 0.0539<br>wR2 = 0.1149
R indices (all data)*	R1=0.0379<br>wR2=0.0812	R1=0.1278<br>wR2=0.2008	R1 = 0.0835<br>wR2 = 0.1162	R1 = 0.0798<br>wR2 = 0.1304
CCDC no.	2062421	2062422	2094997	2094998

* $R1 = \Sigma || Fo| - |Fc|| / \Sigma |Fo|$; $wR2 = = [\Sigma w(Fo^2 - Fc^2)^2 / \Sigma wFo^4]^{½}$

Table S2. Logarithms of stepwise protonation constants of **L2-Me** obtained by ^{1}H NMR measurements and potentiometric measurements (pot.). Values in parentheses are standard deviations in the last significant figure.

**Reaction**	**pK_D (NMR)**	**pK_H (NMR)**	**pK_H (pot.)**
$H_{-1}L^- + H^+ \rightleftarrows H(H_{-1}L)$	12.19(2)	11.37	11.51(2)
$H(H_{-1}L) + H^+ \rightleftarrows H_2(H_{-1}L)^+$	9.47(7)	9.07	8.853(8)
$H_2(H_{-1}L)^+ + H^+ \rightleftarrows H_2L^{2+}$	6.15(7)	5.89	5.718(8)
$H_2L^{2+} + H^+ \rightleftarrows H_3L^{3+}$	1.03(9)	0.99	2.23(4)
$\mathrm{Log}\,\beta = \sum \log K$	28.84(9)	27.32	28.31

Table S3: Bond distances (Å) and angles (°) for the crystal structure of [Cu(H_{-1}**L2-Me**)](ClO_4) (**1**).

**Bond Distances**		**Bond Angles**	
Cu1-O2	1.9238(10)	N1-Cu1-N2	81.52(4)
Cu1-N1	1.9166(9)	N1-Cu1-N3	101.59(4)
Cu1-N2	2.0971(10)	N1-Cu1-N4	82.70(4)
Cu1-N3	2.2351(12)	N2-Cu1-N3	84.17(4)
Cu1-N4	2.0629(11)	N3-Cu1-N4	84.76(4)
Cu2-O1	1.9105(10)	O2-Cu1-N2	95.90(4)
Cu2-N5	1.9268(9)	O2-Cu1-N3	102.72(4)
Cu2-N6	2.0826(9)	O2-Cu1-N4	104.53(4)
Cu2-N7	2.2380(10)	N5-Cu2-N6	81.80(4)
Cu2-N8	2.0931(9)	N5-Cu2-N7	100.32(4)
		N5-Cu2-N8	81.82(4)
		N6-Cu2-N7	84.15(4)
		N7-Cu2-N8	84.86(4)
		O1-Cu2-N6	91.48(4)
		O1-Cu2-N7	92.44(4)
		O1-Cu2-N8	107.67(4)

Table S4: Bond distances (Å) and angles (°) for the crystal structure of [Cu(H_{-1}**L2-Me$_3$**)](ClO_4) (**2**).

Bond Distances		Bond Angles	
Cu1-O2	1.901(2)	N1-Cu1-N2	82.43(10)
Cu1-N1	1.919(2)	N1-Cu1-N3	96.44(10)
Cu1-N2	2.132(3)	N1-Cu1-N4	81.85(10)
Cu1-N3	2.247(3)	N2-Cu1-N3	84.01(10)
Cu1-N4	2.110(3)	N3-Cu1-N4	84.53(10)
		O2-Cu1-N2	99.09(10)
		O2-Cu1-N3	93.50(10)
		O2-Cu1-N4	98.71(10)

Table S5: Bond distances (Å) and angles (°) for the crystal structure of {[(Cu**L2-Me**)(CuH$_{-1}$**L2-Me**)I]·[(Cu**L2-Me**)(CuH$_{-1}$**L2-Me**)]I$_3$}(I$_2$)(I$_5$)$_3$(I$_7$) (**3**).

**Bond Distances**		**Bond Angles**			
Cu1-O2	1.952(8)	O2-Cu1-N2	97.3(3)	N12-Cu3-N10	159.8(4)
Cu1-N2	2.062(9)	O2-Cu1-N4	101.5(3)	N12-Cu3-O4	100.9(4)
Cu1-N4	2.088(9)	O2-Cu1-N3	102.3(4)	N12-Cu3-N11	86.1(4)
Cu1-N3	2.20(1)	O2-Cu1-N1	154.5(4)	N12-Cu3-N9	81.9(4)
Cu1-N1	1.914(9)	N2-Cu1-N4	160.9(4)	N10-Cu3-O4	98.8(4)
Cu2-I27	2.603(2)	N2-Cu1-N3	86.5(4)	N10-Cu3-N11	86.1(4)
Cu2-N5	1.945(9)	N2-Cu1-N1	82.1(4)	N10-Cu3-N9	82.1(4)
Cu2-N7	2.18(1)	N4-Cu1-N3	86.1(4)	O4-Cu3-N11	99.6(3)
Cu2-N6	2.05(1)	N4-Cu1-N1	82.5(4)	O4-Cu3-N9	155.7(4)
Cu2-N8	2.08(1)	N3-Cu1-N1	103.2(4)	N11-Cu3-N9	104.7(4)
Cu3-N12	2.073(9)	I27-Cu2-N5	153.8(3)	I28-Cu4-N13	137.8(4)
Cu3-N10	2.078(9)	I27-Cu2-N7	103.3(3)	I28-Cu4-N16	102.4(3)
Cu3-O4	1.965(8)	I27-Cu2-N6	99.1(3)	I28-Cu4-N14	99.9(3)
Cu3-N11	2.205(9)	I27-Cu2-N8	101.4(2)	I28-Cu4-N15	110.8(5)
Cu3-N9	1.92(1)	N5-Cu2-N7	102.8(4)	N13-Cu4-N16	82.1(4)
Cu4-I28	2.529(7)	N5-Cu2-N6	81.3(4)	N13-Cu4-N14	81.7(4)
Cu4-N13	1.941(9)	N5-Cu2-N8	82.0(4)	N13-Cu4-N15	111.4(5)
Cu4-N16	2.05(1)	N7-Cu2-N6	86.1(4)	N16-Cu4-N14	157.7(4)
Cu4-N14	2.04(1)	N7-Cu2-N8	85.5(4)	N16-Cu4-N15	86.0(4)
Cu4-N15	2.13(1)	N6-Cu2-N8	159.2(4)	N14-Cu4-N15	85.7(4)

Table S6: Bond distances (Å) and angles (°) for the crystal structure of [(Cu**L2-Me$_3$**)(CuH$_{-1}$**L2-Me$_3$**)I]$(I_2)_2(I_5)_2$ (**4**).

Bond Distances		Bond Angles			
Cu1-O1	1.98(1)	O1-Cu1-N7	154.3(6)	N1-Cu2-N2	83.9(9)
Cu1-N7	1.94(2)	O1-Cu1-N6	98.2(6)	N1-Cu2-N3	96.0(8)
Cu1-N6	2.13(2)	O1-Cu1-N8	101.3(6)	N1-Cu2-N4	85.0(8)
Cu1-N8	2.12(2)	O1-Cu1-N5	107.8(6)	N1-Cu2-I13	106.6(6)
Cu1-N5	2.22(2)	N7-Cu1-N6	81.6(7)	N2-Cu2-N3	82.0(8)
Cu2-N1	2.21(2)	N7-Cu1-N8	82.1(7)	N2-Cu2-N4	158.0(8)
Cu2-N2	2.12(2)	N7-Cu1-N5	97.8(7)	N2-Cu2-I13	100.7(6)
Cu2-N3	1.94(2)	N6-Cu1-N8	160.4(6)	N3-Cu2-N4	80.4(7)
Cu2-N4	2.13(2)	N6-Cu1-N5	85.9(6)	N3-Cu2-I13	157.4(6)
Cu2-I13	2.555(3)	N8-Cu1-N5	85.5(6)	N4-Cu2-I13	100.6(5)

Table S7. Bond distances (Å) and angles (°) for iodine molecules and polyiodide anions in compound **3**.

**Bond distances**		**Bond angles**	
I1-I2	2.7779(6)	I1-I2-I3	176.30(5)
I2-I3	3.2212(7)	I2-I3-I4	83.53(4)
I3-I4	3.0496(5)	I3-I4-I5	178.09(1)
I4-I5	2.8230(4)	I10-I9-I8	175.87(1)
I7-I6	2.7816(4)	I9-I8-I7	81.87(1)
I8-I7	3.0928(5)	I8-I7-I6	176.83(2)
I9-I8	3.1543(4)	I11-I12-I13	178.75(2)
I9-I10	2.7799(4)	I12-I13-I14	97.79(3)
I12 -I11	2.8362(5)	I15-I14-I13	174.54(1)
I12-I13	3.0154(6)	I18-I17-I16	173.70(1)
I14-I15	2.7540(6)	I17-I18-I19	94.61(1)
I14-I13	3.1341(6)	I18-I19-I20	177.98(2)
I17-I16	2.7699(6)	I26-I25-I27	176.20(4)
I17-I18	3.1179(6)		
I19-I18	3.0763(6)		
I19-I20	2.8108(5)		
I25-I26	2.7537(5)		
I27 -I25	3.2370(6)		
I21-I22	2.7286(5)		
I24-I23	2.7355(5)		

Table S8. Bond distances (Å) and angles (°) for iodine molecules and polyiodide anions in compound **4**.

**Bond distances**		**Bond angles**	
I1-I2	2.767(3)	I1-I2-I3	173.96(8)
I2-I3	3.128(3)	I2-I3-I4	122.96(7)
I3-I4	3.187(3)	I3-I4-I5	178.22(8)
I4-I5	2.772(3)	I10-I9-I8	177.57(9)
I6-I7	2.733(2)	I9-I8-I15	96.07(6)
I10-I9	2.776(3)	I8-I15-I14	173.67(7)
I9-I8	3.116(2)	I11-I12-I13	165.93(9)
I8-I15	3.252(2)		
I15-I14	2.738(2)		
I11-I12	2.753(3)		
I13-I12	3.361(3)		

Table S9. Percentual breakdown of Hirshfeld surface contribution by element for cases illustrated in Fig. 12.

Inside Atom	Outside Atom						
Fig. 16a	I	Cu	O	H	N	C	Total
C	4.3	.	.	1.6	.	.	5.9
Cu	0.6	.	.	0	.	.	0.6
H	54.6	.	2.6	23.7	.	0.8	81.7
I	2	.	.	4.9	.	.	6.9
N	1	.	.	0	.	.	1
O	0.6	.	0	3.3	.	.	4
Total	63.1	0	2.6	33.6	0	0.8	
Fig. 16b							
C	5.1	.	0	1.5	.	.	6.7
Cu	0	.	.	.	.	.	0
H	54.1	.	2.7	23.7	.	0.8	81.3
I	0.5	.	.	6.8	.	.	7.3
N	0.8	.	.	.	.	.	0.8
O	0.5	.	0	3.4	.	.	3.8
Total	61.1	0	2.7	35.4	0	0.8	
Fig. 16c							
C	3	.	.	0.8	.	.	3.8
Cu	0.6	.	.	.	.	.	0.6
H	57.6	.	3.1	24.9	.	0.4	86
I	1.6	.	.	3.4	.	.	5
N	0.9	.	.	.	.	.	0.9
O	0.2	.	.	3.5	.	.	3.7
Total	63.9	0	3.1	32.6	0	0.4	

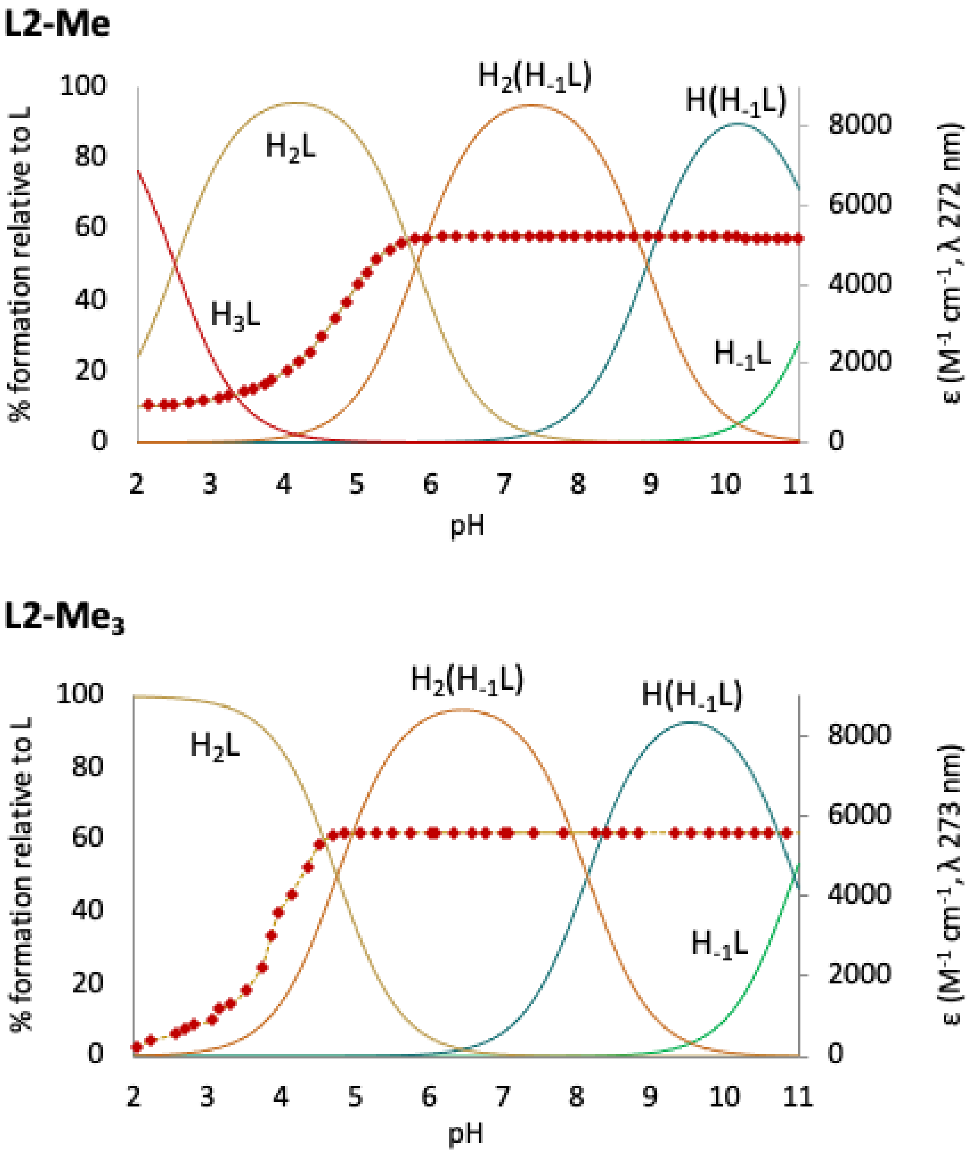

Figure S1. Distribution diagrams of the protonated species formed by **L2-Me** and **L2-Me₃** as a function of pH in aqueous solution. The extinction coefficient associated to the pyridine band in the UV-Vis spectra is represented as red diamonds (♦).

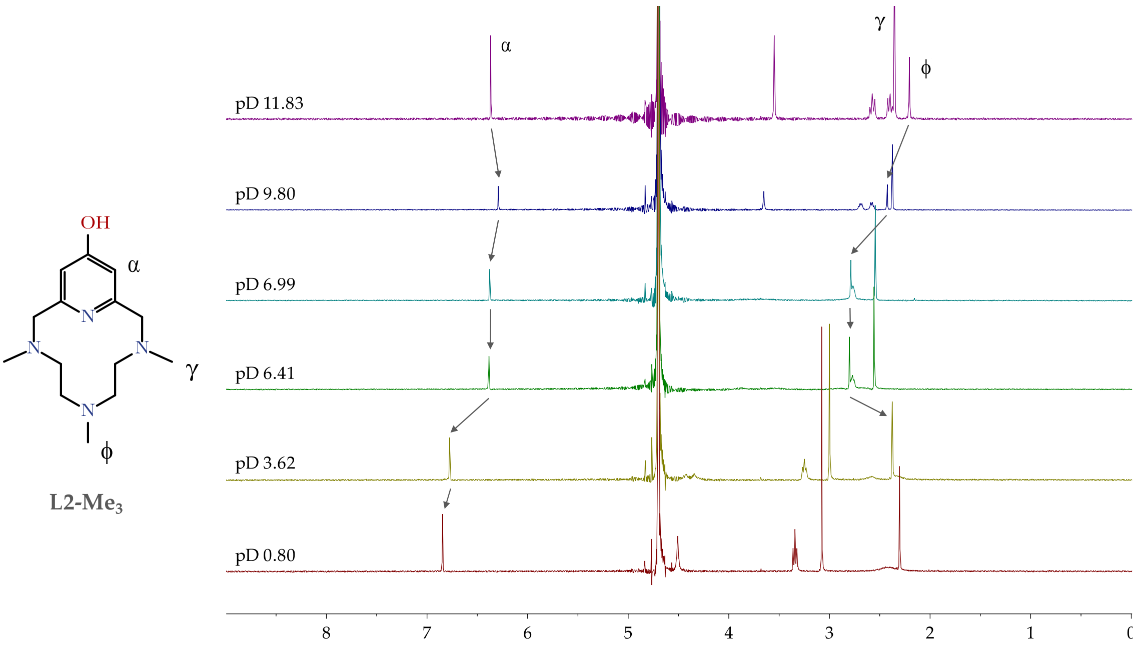

Figure S2. Overlay of the ^{1}H-NMR spectra of **L2-Me$_3$** measured in D_2O solution from pD 0.80 to 11.83.

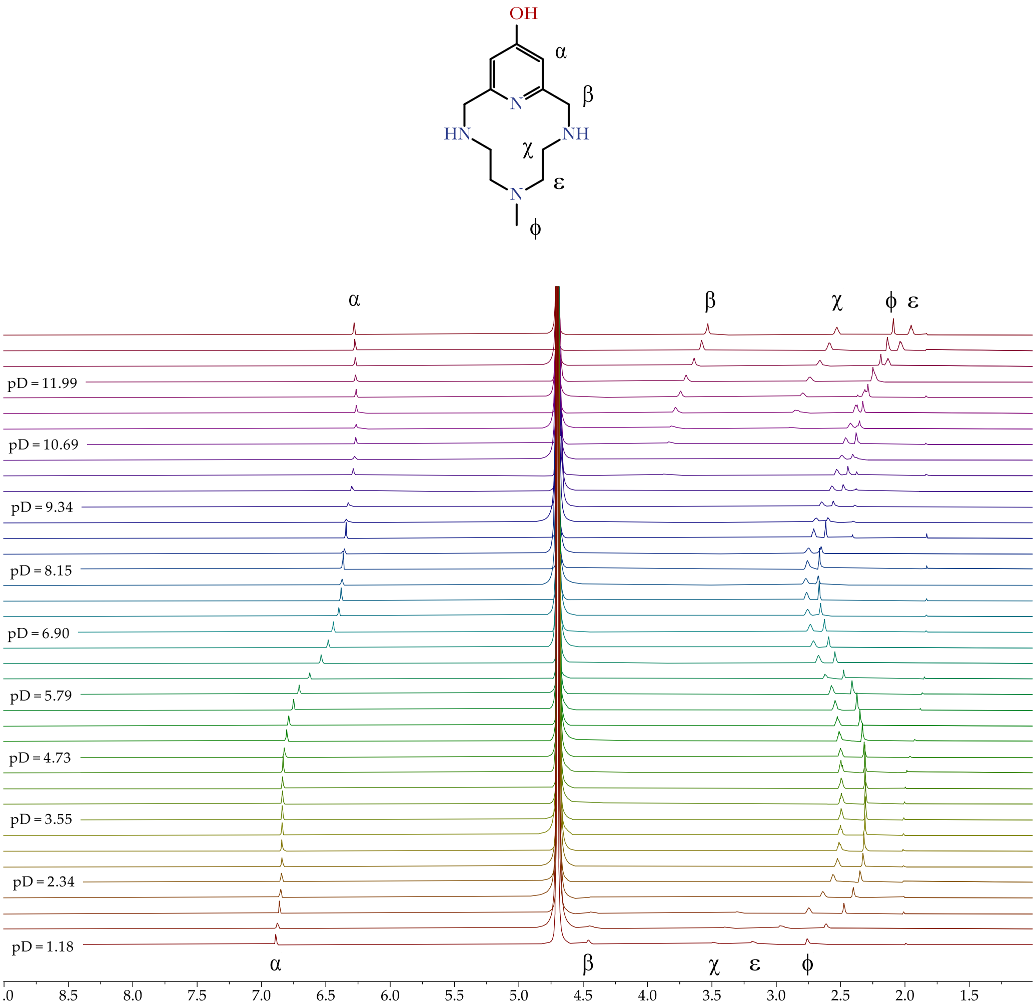

Figure S3. Overlay of the ^{1}H-NMR spectra of **L2-Me** measured in D_2O solution from pD 1.18 to 13.02.

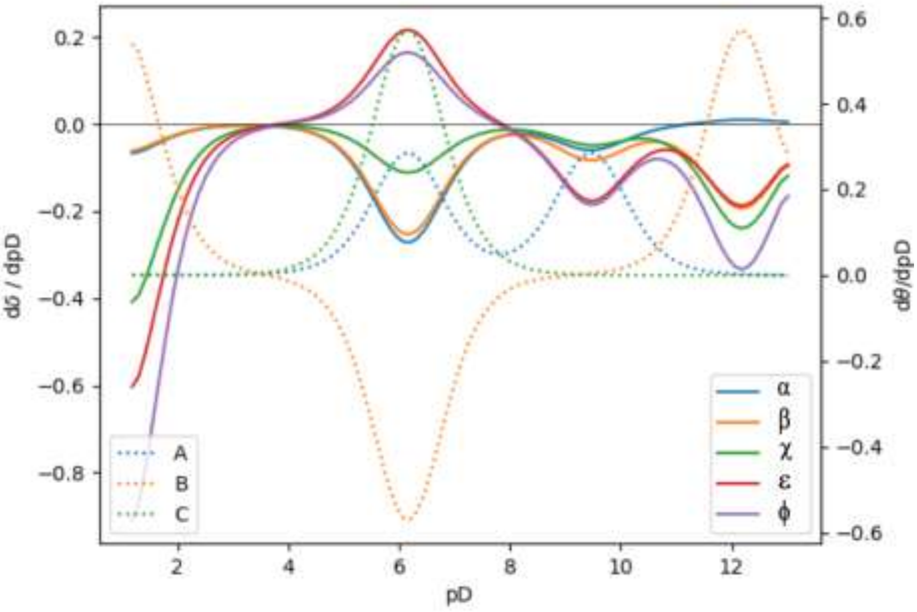

Figure S4. Derivative of calculated chemical shifts with respect to pD (left axis, solid lines). Derivative of a protonation site occupancy (theta) with respect to pD (right axis, dashed lines).

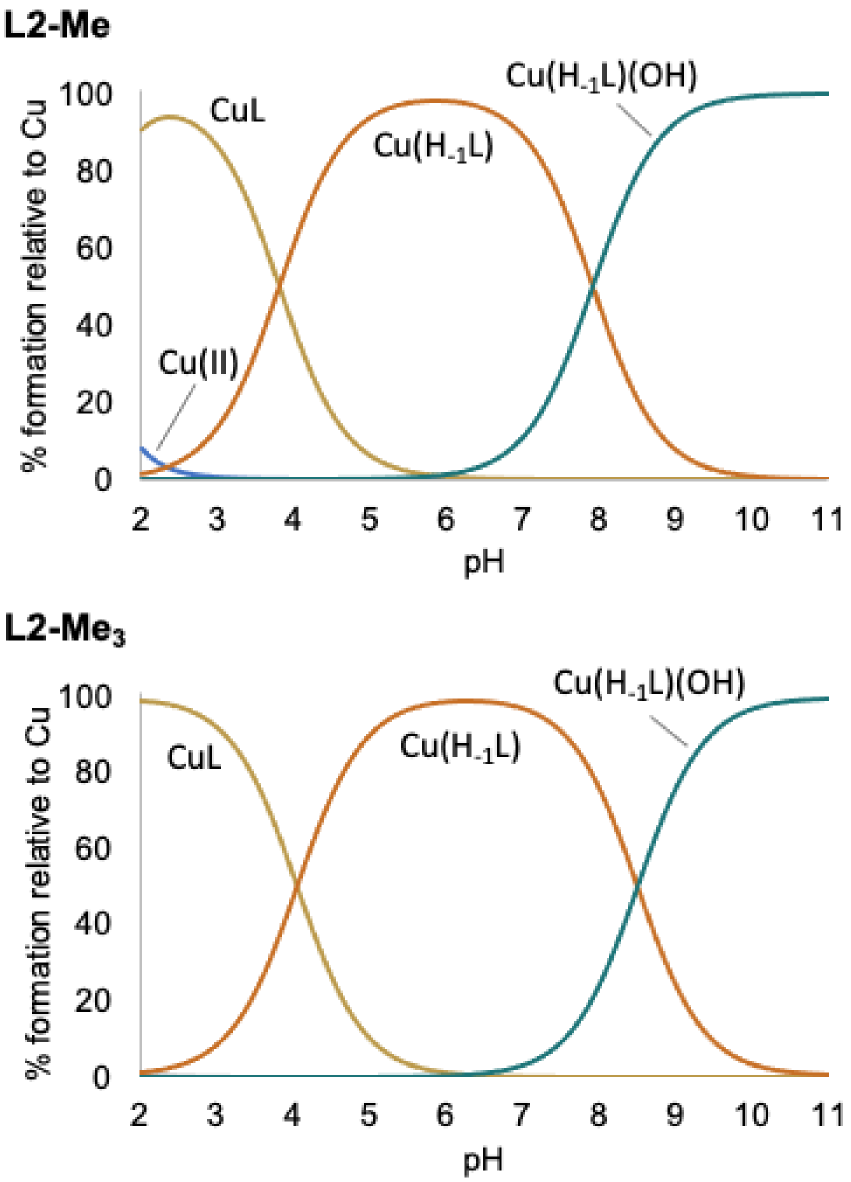

Figure S5. Distribution diagrams of the complexes formed in the systems Cu(II)/**L2-Me** and Cu(II)/**L2-Me$_3$**. [Cu(II)] = [ligand] = 1 mM. Charges omitted.

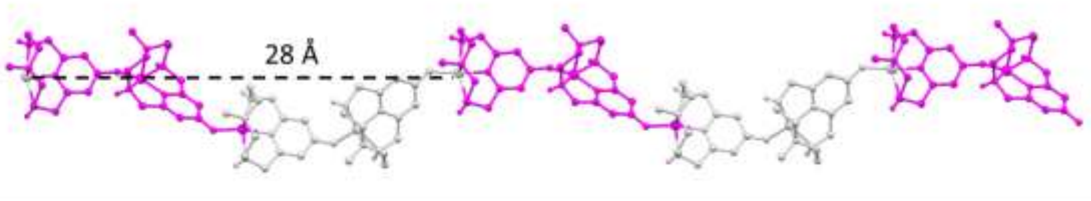

a

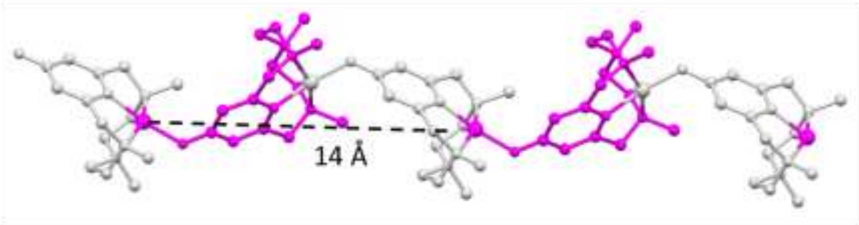

b

Figure S6. Helical arrangement of the coordination polymers in (a) [Cu(H_{-1}**L2-Me**)](ClO_4)·0.716H_2O (**1**) and (b) [Cu(H_{-1}**L2-Me₃**)](ClO_4)·H_2O (**2**).

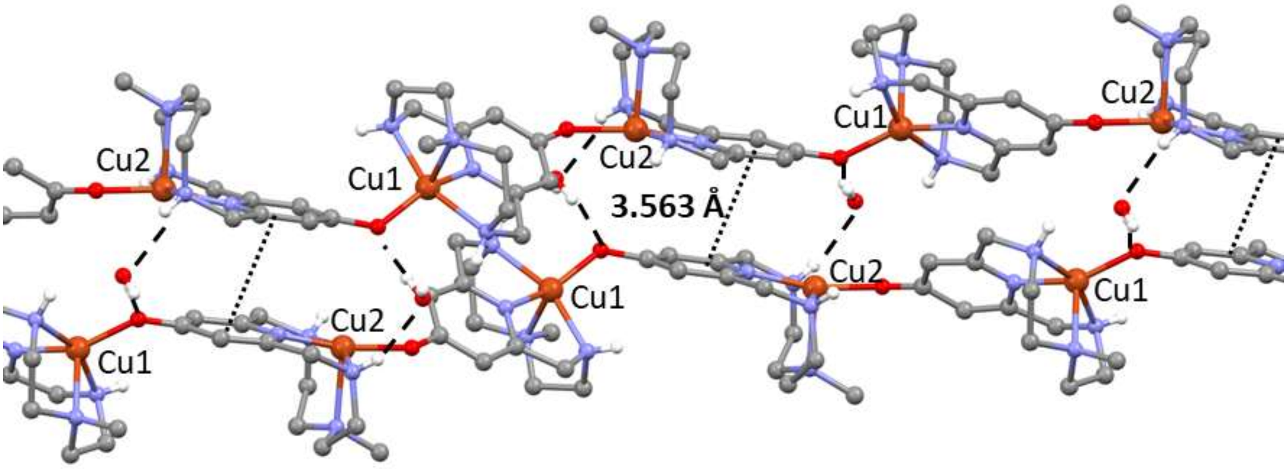

Figure S7. View of a pair of polymeric chains found in of [CuH$_{-1}$**L2-Me**](ClO_4)·0.716H_2O (**1**).

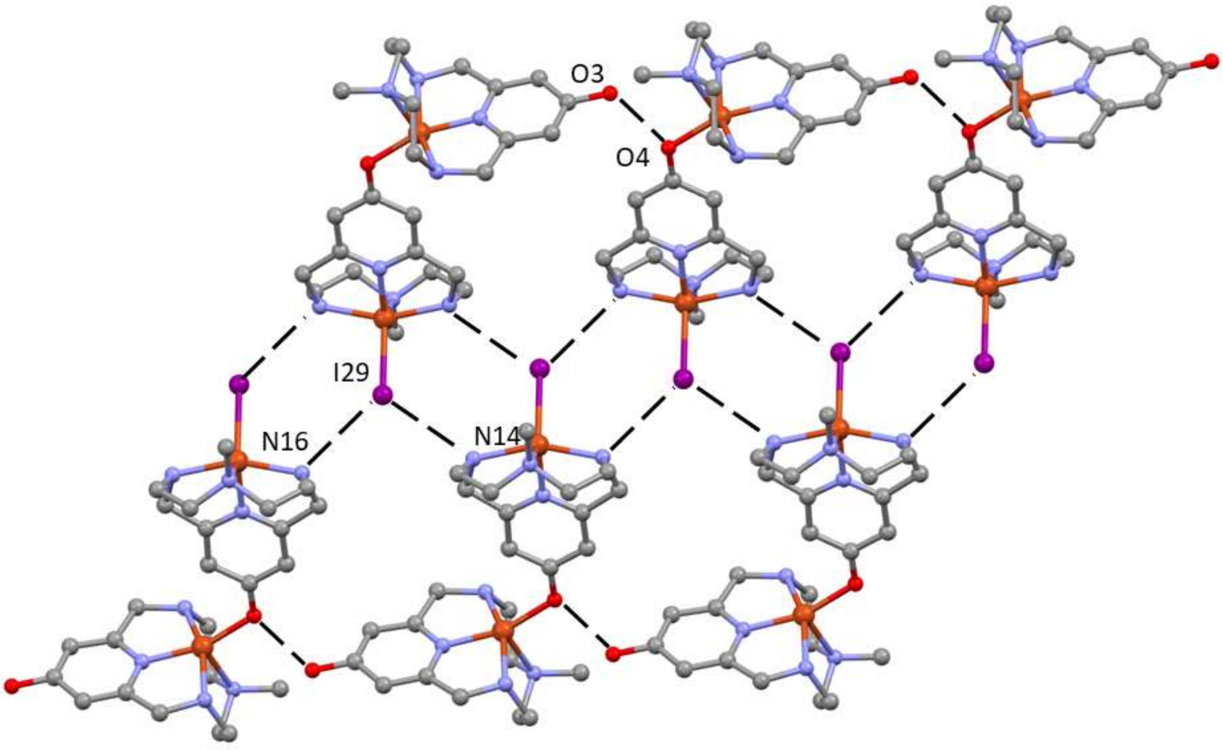

Figure S8. Array, growing along the b axis, of $[(Cu\mathbf{L2\text{-}Me})(CuH_{-1}\mathbf{L2\text{-}Me})I]^{2+}$ binuclear complexes linked by charge assisted $OH\cdots O^-$ and $NH\cdots I^-$ H-bonds in the I29 minor component.

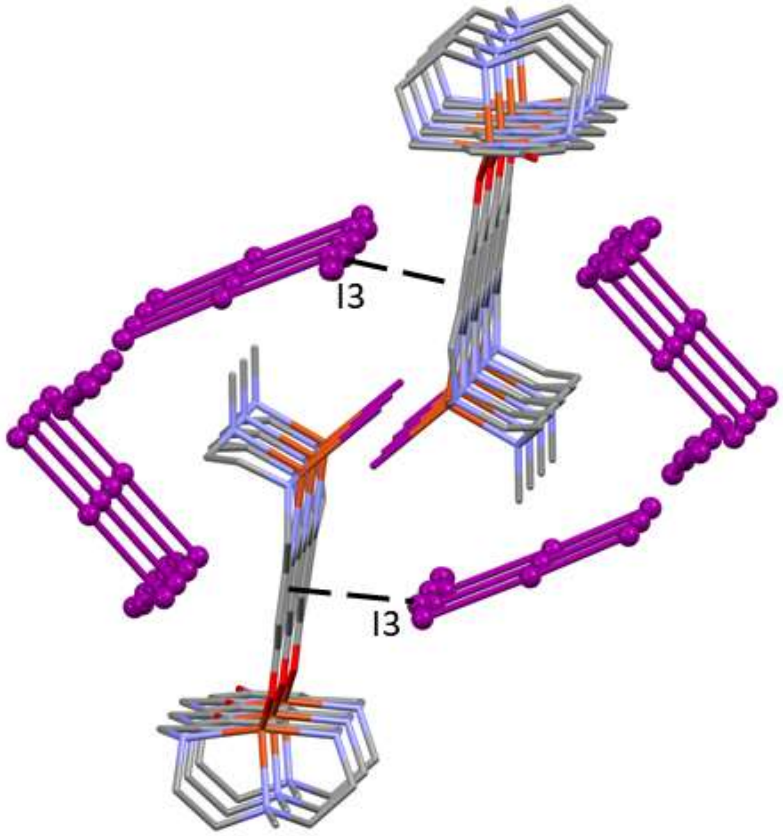

Figure S9. Compound **3.** Array, growing along the b axis, of [(Cu**L2-Me**)(CuH$_{-1}$**L2-Me**)I]$^{2+}$ binuclear complexes surrounded by tapes of triiodide anions and iodine molecules. Anion-π interaction also involving the pyridinol ring of (CuH$_{-1}$**L2-Me**)I.

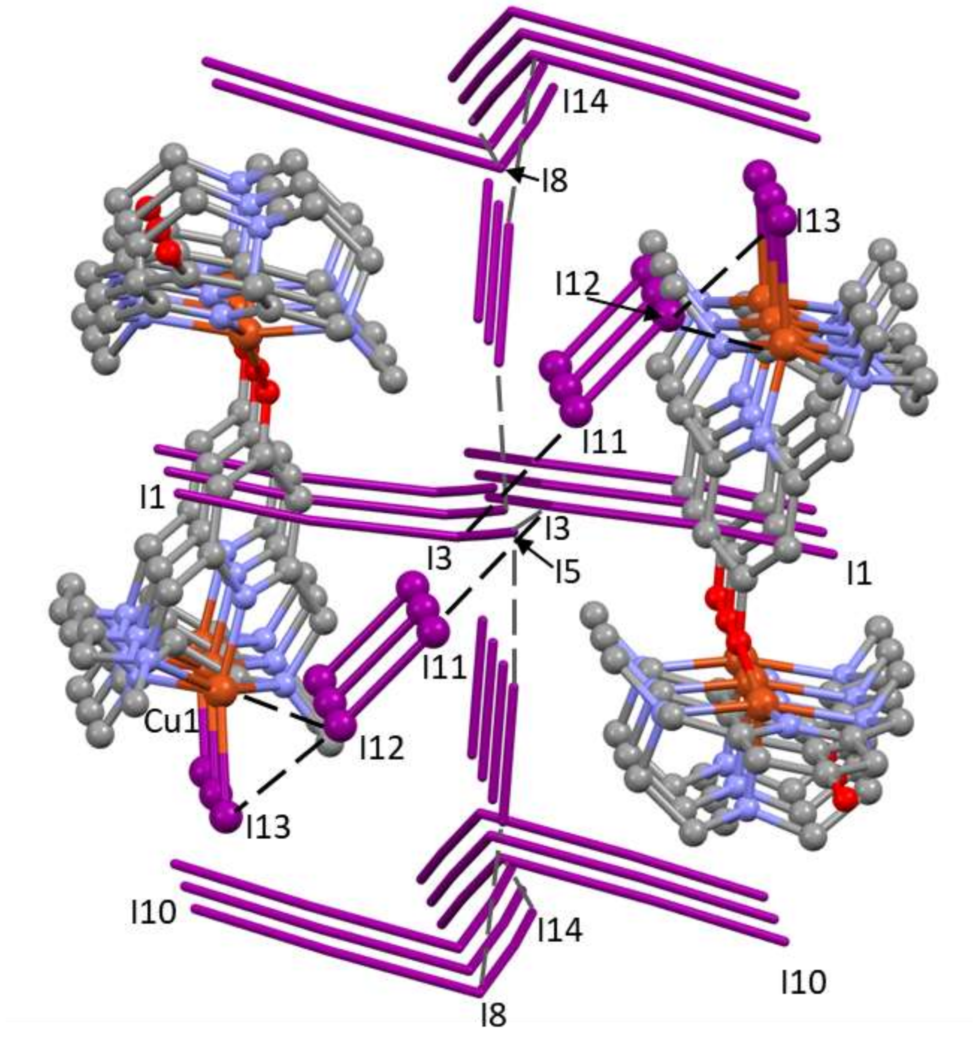

Figure S10. Compound **4**. Contacts established between the grids' pentaiodide atoms and the iodine molecules weakly interacting with Cu1.

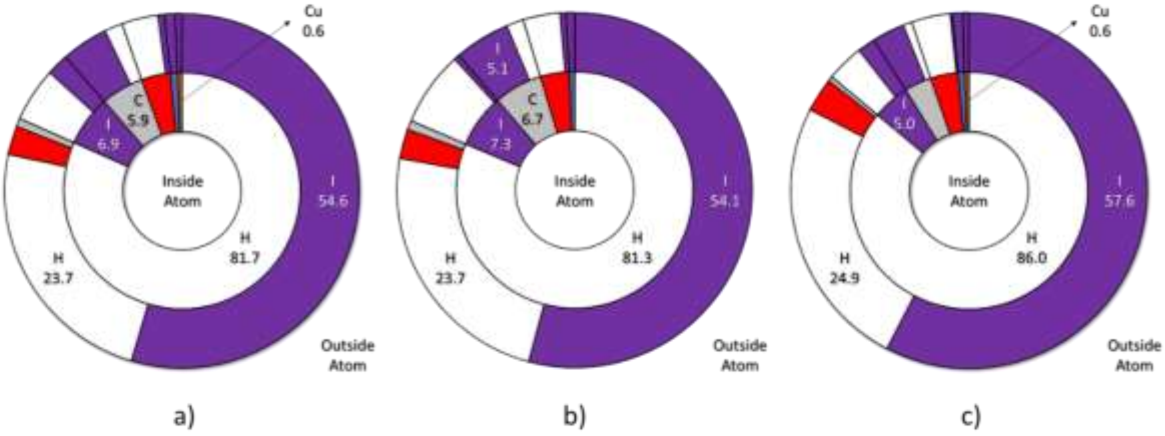

Figure S11. Overview of Hirschfeld surface composition for polyiodide crystal structures: a) **3**, surface of $[(CuL)(CuH_{-1} L)I]^{2+}$ (L=**L2-Me)** formally coordinated by I_3^-; b) **3**, surface of $[(CuL)(CuH_{-1} L)I]^{2+}$ (L=**L2-Me)** formally coordinated by I^-; c) **4**, surface of $[(CuL)(CuH_{-1} L)I]^{2+}$ (**L2-Me$_3$)**. Inner ring: internal composition of Hirschfeld surface, i.e. a depiction of $[(CuL)(CuH_{-1}L)I]^{2+}$ (L= **L2-Me or L2-Me$_3$)** molecular surface; outer rings: closest outer atom, i.e. atom in contact with $[(CuL)(CuH_{-1}L)I]^{2+}$ (L= **L2-Me or L2-Me$_3$)** Hirschfeld surface. Numerical values reported only for components ≥5%. Complete data in tabular form are found in Table S6.

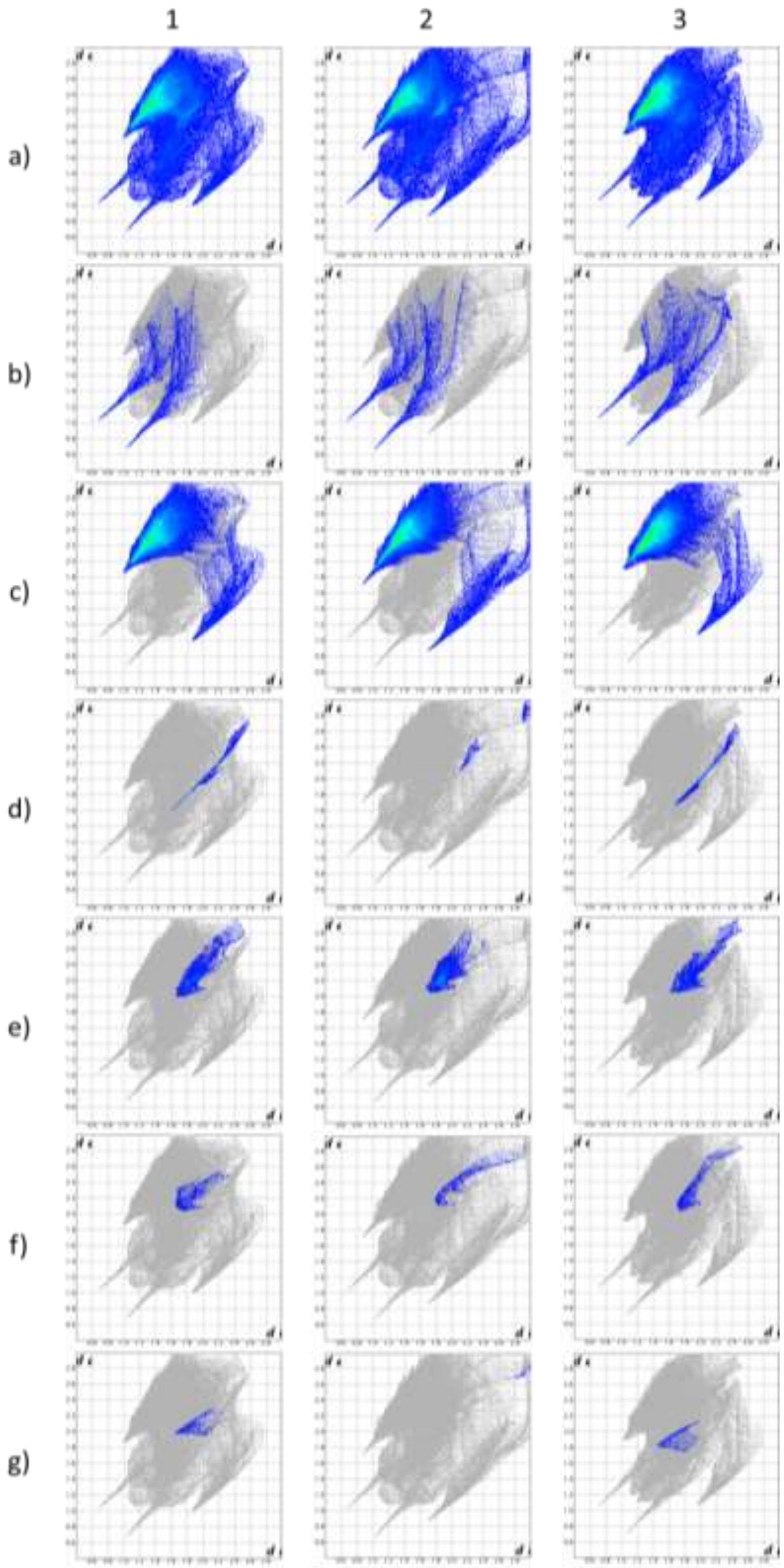

Figure S12. Overview of fingerprint plots features for: **3**, surface of $[(CuL)(CuH_{-1} L)I]^{2+}$ formally coordinated by I_3^- (1); **3**, surface of $[(CuL)(CuH_{-1} L)I]^{2+}$ formally coordinated by I^- (2); **4**, surface of $[(CuL)(CuH_{-1} L)I]^{2+}$ (3). Plot shows: a) global fingerprint; b) O···H and reciprocal contacts; c) I···H and reciprocal contacts; d) I···I contacts; e) C···I and reciprocal contacts; f) N···I and reciprocal contacts; g) Cu···I contacts.

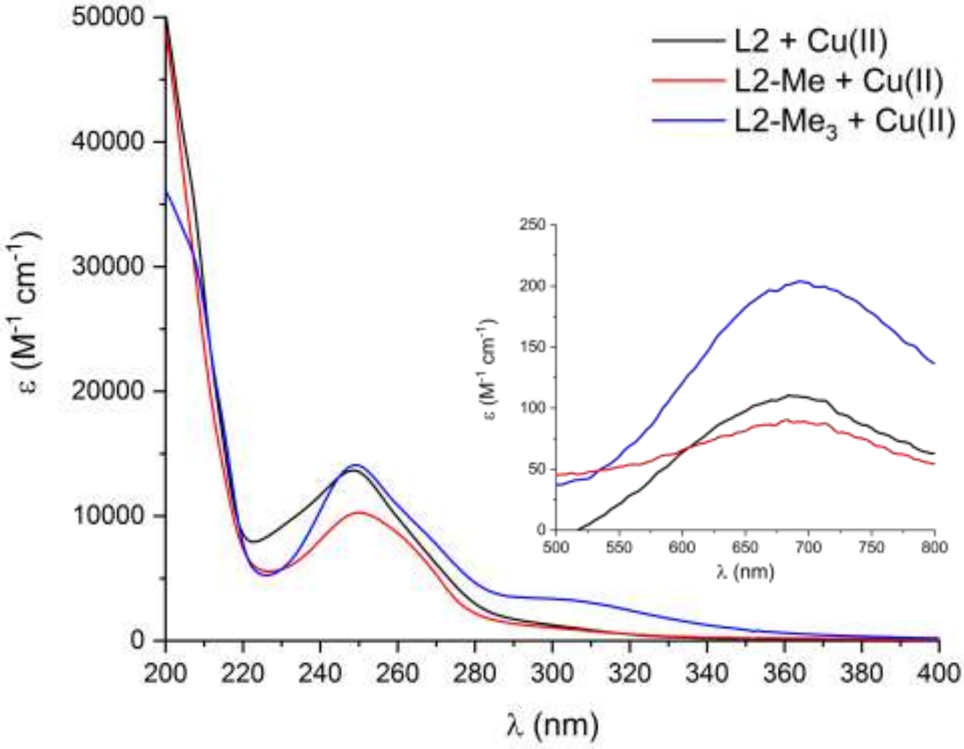

Figure S13. Uv-vis spectra of $[CuH_{-1}\mathbf{L2}]^+$, $[CuH_{-1}\mathbf{L2\text{-}Me}]^+$ e $[CuH_{-1}\mathbf{L2\text{-}Me_3}]^+$ recorded in aqueous solution at pH 7.5, 6.0 and 6.5, respectively.

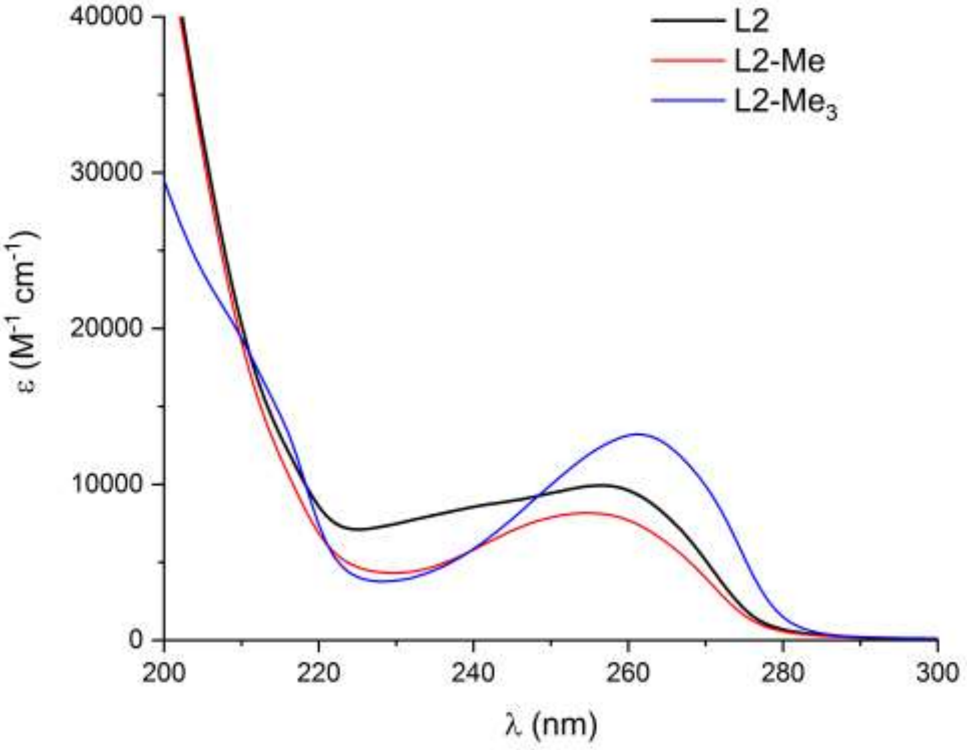

Figure S14. Uv-vis spectra of **L2**, **L2-Me** and **L2-Me$_3$** recorded in aqueous solution at pH 7.5, 6.0 and 6.5, respectively.

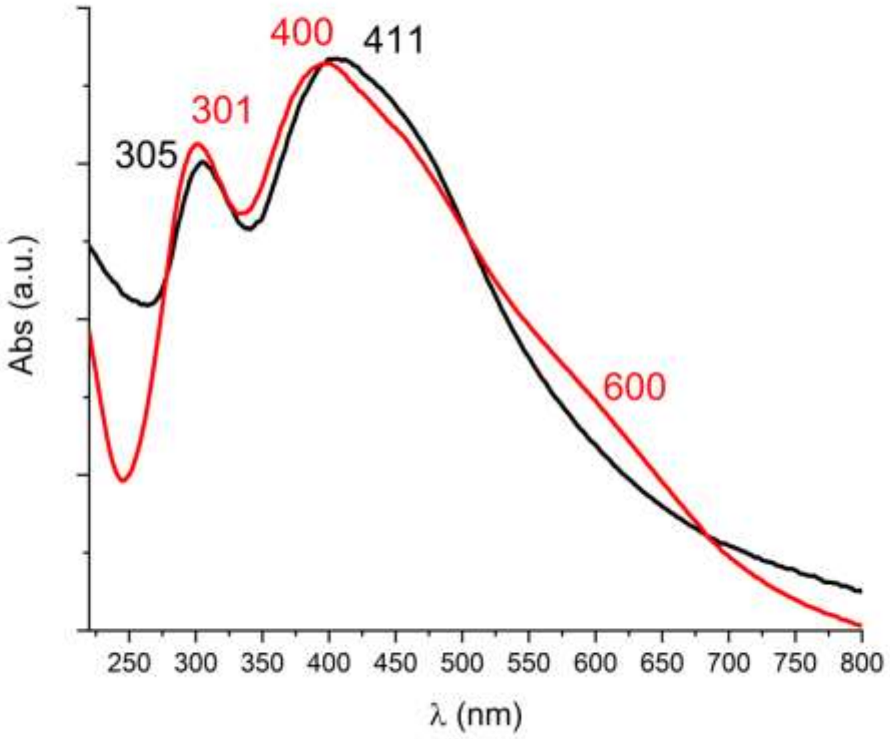

Figure S15. UV-vis spectra of thin films of {[(Cu**L2-Me**)(CuH$_{-1}$**L2-Me**)I]·[(Cu**L2-Me**)(CuH$_{-1}$**L2-Me**)]I_3}$(I_2)(I_5)_3(I_7)$ (**3**) (black) and [(Cu**L2-Me$_3$**)(CuH$_{-1}$**L2-Me$_3$**)I]$(I_2)_2(I_5)_2$ (**4**) (red).
